# Bioactive Compounds from Plant Origin as Natural Antimicrobial Agents for the Treatment of Wound Infections

**DOI:** 10.3390/ijms25042100

**Published:** 2024-02-08

**Authors:** Katarzyna Pacyga, Paweł Pacyga, Ewa Topola, Szymon Viscardi, Anna Duda-Madej

**Affiliations:** 1 Department of Environment Hygiene and Animal Welfare, Faculty of Biology and Animal Science, Wroclaw University of Environmental and Life Sciences, 50-375 Wroclaw, Poland; 2 Department of Thermodynamics and Renewable Energy Sources, Faculty of Mechanical and Power Engineering, Wrocław University of Science and Technology, 50-370 Wrocław, Poland; pawel.pacyga@pwr.edu.pl; 3 Faculty of Medicine, Wroclaw Medical University, Ludwika Pasteura 1, 50-367 Wrocław, Poland; ewa.topola@student.umw.edu.pl (E.T.); szymon.viscardi@student.umw.edu.pl (S.V.); 4 Department of Microbiology, Faculty of Medicine, Wroclaw Medical University, Chałubińskiego 4, 50-368 Wrocław, Poland

**Keywords:** plants, bioactive compounds, antibacterial activity, molecular effects, drug-resistant bacteria

## Abstract

The rising prevalence of drug-resistant bacteria underscores the need to search for innovative and nature-based solutions. One of the approaches may be the use of plants that constitute a rich source of miscellaneous compounds with a wide range of biological properties. This review explores the antimicrobial activity of seven bioactives and their possible molecular mechanisms of action. Special attention was focused on the antibacterial properties of berberine, catechin, chelerythrine, cinnamaldehyde, ellagic acid, proanthocyanidin, and sanguinarine against *Staphylococcus aureus*, *Enterococcus* spp., *Klebsiella pneumoniae*, *Acinetobacter baumannii*, *Escherichia coli*, *Serratia marcescens* and *Pseudomonas aeruginosa*. The growing interest in novel therapeutic strategies based on new plant-derived formulations was confirmed by the growing number of articles. Natural products are one of the most promising and intensively examined agents to combat the consequences of the overuse and misuse of classical antibiotics.

## 1. Introduction

Inflammation is a complex disease process caused by endo- or exogenous factors, which can be biological (e.g., bacteria, viruses, fungi, protozoa, endotoxins, exotoxins), physical (e.g., mechanical, ionising radiation, magnetic field, ultrasound waves), or chemical (e.g., acids, bases) [1]. In the process of an inflammatory reaction, cells of the immune system (e.g., macrophages, neutrophils, monocytes, lymphocytes, mast cells), connective tissue cells (e.g., eicosanoids, i.e., prostaglandins, leukotrienes, and lipoxins), certain blood proteins (e.g., complement components, kinins, histamine, serotonin), and blood vessels are involved [2]. Additionally, adhesion molecules, responsible for interactions between leukocytes and endothelial cells, are involved in the pathomechanism of the inflammatory response. These include selectins (P and E-present on the epithelial surface, L-present on the surface of leukocytes), immunoglobulins (ICAM-intercellular, VCAM-vascular), integrins, and mucin-like glycoproteins [3,4]. The expression of these adhesion molecules is dependent on the presence of inflammatory mediators in the environment, i.e., histamine, platelet activating factor (PAF), IL-1, and TNF-α [5]. Recognition of the relevant adhesion molecules initiates the body’s response to the inflammatory agent, which can take the form of (1) cellular (T-lymphocyte involvement)-directed mostly against intracellular pathogens, (2) humoral (B-lymphocyte involvement)-protecting mainly against extracellular microorganisms, and (3) hemostatic (blood component involvement)-leading to platelet aggregation, clot formation, and intravascular coagulation.

The inflammatory response process is usually characterised by a dynamic and short-lived pattern that stops after the causative agent is demarcated and inactivated, as well as the regeneration of the damaged tissue. However, when the anti-inflammatory response is prolonged, ineffective, or excessive, chronic inflammation develops. In its course, the stages of inflammation, i.e., tissue damage, an increase in blood flow, the influx of immune cells, and the repair process, occur simultaneously. The result of the response to chronic immune cell activity is abnormal tissue remodelling, irreversible tissue damage, and the development of a number of chronic diseases [2,6,7]. Inflammation has long been recognised as the cause of various diseases. Tissue damage in the chronic course of the inflammatory response is observed not only in inflammatory diseases but also with ongoing cancer. This includes, for example, the heart [8,9], pancreas [10,11,12], liver [13], lungs [14], kidneys [15], gastrointestinal tract [16,17], genital tract [18], and also the brain [19,20]. The situation can become dramatic when the cause of inflammation is bacteria, especially those that produce toxins. Bacterial toxins and their producers, through successive events, can lead to a dramatic inflammatory response in the body—sepsis [21]. It is an immediate, life-threatening situation associated with the breakdown of many critical organs, such as the kidneys, liver, heart, and lungs.

Over the past half-century, antibiotics have become the basis for the eradication of infectious diseases. They have found use in the prevention of infections of Gram-positive, Gram-negative, and atypical bacterial etiologies. However, despite progress in diagnosis and therapy, these diseases are still the major cause of death worldwide [22]. Despite the fact that the incidence of infectious diseases has been on an increasing trend from year to year, since the COVID-19 pandemic, the situation seems catastrophic. Recently, there has been an increase not only in viral, i.e., RSV [23], chickenpox, monkeypox [24], and measles [25], but especially bacterial ones, i.e., scarlet fever (*Streptococcus pyogenes*) [26], pertussis (*Bordetella pertussis*) [27], syphilis (*Treponema pallidum*) [28,29], and gonorrhoea (*Neisseria gonorrhoeae*) [29] infections. A large part of this increase is the result of “immune debt”, a situation in which the body acquires immunity through outbreeding, which was lower during the pandemic. However, this state has undoubtedly been further aided by popular anti-vaccine groups and the problem of emigration [30,31,32,33]. In addition, the overuse of drugs observed in recent years has certainly contributed to the whole reality. The prevalence of antibiotics in the environment, e.g., use in veterinary medicine, agriculture, and animal nutrition, as well as unjustified use and inappropriate dosage, i.e., in viral infections and bacterial carriage, has also become an additional factor [34]. This has led to the emerging resistance of bacterial strains to drugs of last resort, i.e., carbapenems, glycopeptides, or colistin [35,36,37,38]. The above behaviours have created a huge threat to the effectiveness of eradicating bacterial etiological agents in the treatment of infections.

Bacterial resistance is related to the adaptation and survival of the microorganism to a new environment in the presence of an antibiotic. Bacteria can: (1) block the penetration of a drug into a bacterial cell (e.g., resistance to β-lactam antibiotics, tetracyclines); (2) prevent the drug from reaching its target site in the cell (e.g., resistance to fluoroquinolones, tetracyclines); (3) modify the drug’s target site (e.g., resistance to β-lactam antibiotics, fluoroquinolones, macrolides, aminoglycosides, glycopeptides); and (4) inactivate or alter the drug by producing special enzymes (e.g., resistance to β-lactam antibiotics, aminoglycosides, chloramphenicol) [39]. In most cases, resistance is genetically determined. Spontaneous point mutations and recombination of genetic material can lead to resistant strains. In addition, this material can be transferred between microorganisms by (1) transformation (acquisition and incorporation of a free DNA fragment into the genome), (2) transduction (introduction of resistance genes by a bacteriophage), and (3) conjugation (transfer of plasmid-extra-chromosomal genetic material and transposons-fragments of DNA capable of movement) [40]. In these ways, resistance genes, not only between pathogenic bacteria but also their reservoirs, can become representatives of the microbiome.

In order to prevent the 21st century from entering history as a post-antibiotic era, it becomes necessary to look for new solutions. These are found in the products that nature is providing us. Plants contain a wide variety of valuable substances that exhibit a range of biological activities, such as antioxidant, antibacterial, and antifungal. They can restore the clinical use of some older antibiotics by increasing their effectiveness and thus avoiding resistance development [41]. According to the WHO, the urgency to develop new drugs lists the highest-risk group, which includes (1) Gram-negative bacteria, i.e., *Pseudomonas aeruginosa* and *Acinetobacter baumannii* carbapenem-resistant; (2) *Enterobacterales* producing β-lactamases with an extended substrate spectrum (ESBLs) and resistant to carbapenems; and furthermore (3) *Mycobacterium tuberculosis* complex (MBTC) rifampicin-resistant. In addition, many reports include infections with *Neisseria gonorrhoeae*, which is reported to be resistant to all antibiotics in various parts of the world [42]. The next group includes strains of (1) methicillin- and vancomycin-resistant *Staphylococcus aureus* and (2) vancomycin- and linezolid-resistant *Enterococcus* spp. In turn, the rest of the groups include pathogens responsible for out-of-hospital infections for which no oral antibiotic is available for therapy (due to a lack of these antibiotics) [43].

The aim of this review was to provide a comprehensive summary of the antibacterial potential of seven bioactive compounds (berberine, catechin, chelerythrine, cinnamaldehyde, ellagic acid, proanthocyanidins, and sanguinarine) from plant origin against *Staphylococcus aureus*, *Staphylococcus epidermidis*, *Enterococcus* spp., *Klebsiella pneumoniae*, *Acinetobacter baumannii*, *Escherichia coli*, and *Pseudomonas aeruginosa*. In addition, the brief characteristics and biological activities of these substances were introduced. The most recent findings, from 1999 to 2023, on this topic were highlighted.

## 2. Results and Discussion

In this review, seven plant-delivered compounds (berberine, catechin, chelerythrine, cinnamaldehyde, ellagic acid, proanthocyanidin, and sanguinarine) that have great potential for the production of natural antibiotics have been discussed.

### 2.1. Berberine (BRB)

**Figure 1:**
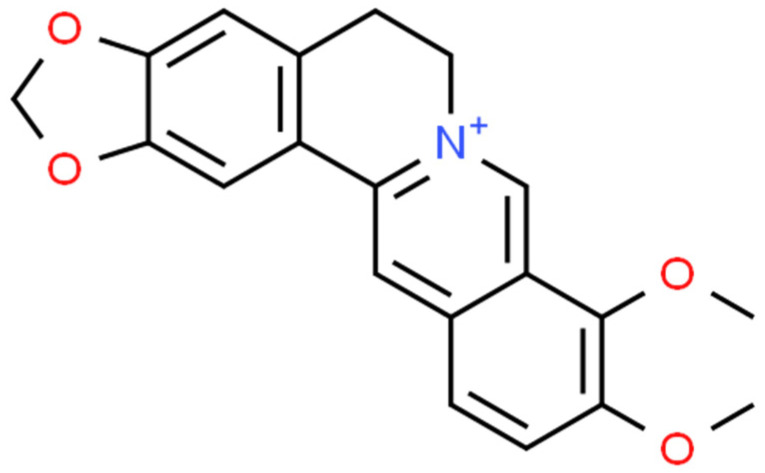
Berberine structure (ChemSpider database).

**Figure 2:**
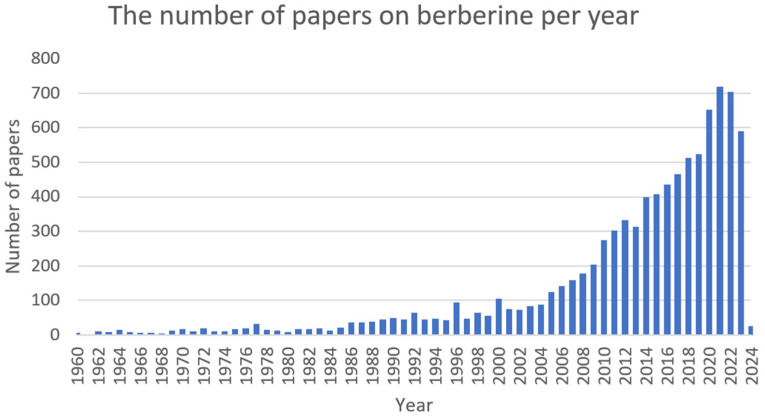
The number of papers on berberine per year (PubMed database).

Berberine (Figure 1) is a plant metabolite that belongs to the group of isoquinoline alkaloids and exhibits low toxicity as well as strong biological and pharmacological activities [44,45]. It is a very important natural alkaloid that is used to synthesise various bioactive derivatives by modifying functional groups that are important for the development of new and selective treatments [46]. Among the *Berberidaceae* family, the genus *Berberis* includes approximately 450–500 species, which are the major natural sources of berberine [45,46]. This active compound can be found in the leaves, stems, twigs, barks, rhizomes, and roots of many medicinal plants [46]. At present, berberine is of great interest due to its, among others, anticancer [47,48], cholesterol-lowering [49], antidiabetic [50], anti-obesity [51], hypolipidemic [52], hepatoprotective [53], antihypertensive [54], antidepressant [55], anti-inflammatory [56], antidiarrheal [57], anti-neurological [58,59], antibacterial [60], and antiviral [61] properties. However, the favourable applications of berberine are restricted by the weak pharmacokinetics of the compound. Nonetheless, berberine appears to be very effective and can be used in combination with other chemotherapeutics or treatments [44]. The growing interest in this compound can be seen in the increasing number of articles (Figure 2).

#### 2.1.1. Antimicrobial Activity

##### *Staphylococcus* *aureus*

The study by Sahibzada et al. [62] revealed that berberine nanoparticles (BRB NPs) with enhanced bioavailability could be used as potential antibacterial and antifungal agents. Their activity was tested in an in vitro assay against Gram-negative (*Escherichia coli*, *Pseudomonas aeruginosa*) and Gram-positive (*S. aureus*, *Bacillus subtilis*). The research demonstrated that BRB NPs exerted the highest activity in comparison to unprocessed BRB, as well as norfloxacin and clarithromycin. Nanoparticles developed by the EPN (evaporative precipitation of nanosuspension) method showed 3–4 times higher activity against Gram-negative bacteria than unprocessed berberine, while NPs developed by the APSP (anti-solvent precipitation with a syringe pump) method presented 2–3 times higher activity against Gram-positive pathogens. These findings are of great importance, as previous studies on berberine showed its lower activity against gram-positive (*S. aureus*) and gram-negative (*E. coli*) bacteria (MIC ≥ 512 μg·mL^−1^; minimum inhibitory concentration) [62]. Therefore, novel approaches and the development of innovative nanoparticles that increase the bioactivity of natural compounds are crucial [63].

Zhang CF et al. (2022), in their study, presented the impact of berberine on *S. aureus* (NCTC8325) biofilm formation and proposed the molecular basis of this process. The observed MIC value amounted to 256 μg·mL^−1^. The hindered dispersion of *S. aureus* was noted at a concentration of 32 μg·mL^−1^ [64]. The effectiveness of BRB nanoparticle complexes with epigallocatechin gallate (EGC) against MDR (multidrug-resistant) *S. aureus* has also been demonstrated. The applied combination showed low systemic toxicity. Furthermore, the in vivo murine model for the study of MRSA (methicillin-resistant *S. aureus*) infection revealed that BRB-EGC complexes had a high bacteriostatic efficacy, even higher than benzylpenicillin (BP) or ciprofloxacin (CYP). Moreover, the in vivo studies demonstrated that the combination of BRB and EGC has the potential to enhance wound healing in mice infected with MRSA when compared to BRB alone, CYP, or BP [65].

An interesting study of the antimicrobial activity of berberine against clinical-derived PJI (prosthetic joint infection)-related *S*. *aureus* was evaluated. This study is vitally crucial, as it is the most devastating complication after arthroplasty surgeries. The authors found that the MIC values varied from 64 to 512 µg·mL^−1^ among multi-locus sequence types, and the bacteria were able to regain growth after 24 h in BRB of MIC value or higher concentrations. What is also intriguing is that the experiment conducted showed that the sub-inhibitory concentrations of berberine increased biofilm formation in several *S*. *aureus* strains [66].

The synergistic effect of BRB with sodium houttuyfonate (SH) against MRSA and VISA (vancomycin-intermediate *S. aureus*) was demonstrated in the study conducted by Li et al. (2020). The researchers reported a decrease in the MIC value of BRB from 4 to 64 times in combination with SH. Due to the absence of an effective drug against VISA, both compounds could be an intriguing therapeutic approach [67]. It was shown that the BRB application can also affect the ability of MRSA to create a biofilm. The molecular detection revealed that BRB induces the inhibition of *agrA-D* gene expression, which is responsible for the formation of biofilm in MRSA (the role of quorum-sensing, QR, inhibitor). The MIC and the MBC (minimum bactericidal concentration) values for BRB were 0.05 µg·mL^−1^ and 0.1 µg·mL^−1^, respectively [68]. Tan et al. (2019) presented the results of an in vitro study on the use of BRB as an antimicrobial agent against *S. aureus* causing infection of joint prostheses. The MIC value for BRB ranged between 64 and 512 μg·mL^−1^. Efficacy against planktonic forms of bacteria as well as a reduction in biofilm formation were observed. According to the authors, due to its proven synergistic effect with various antibiotics, berberine can be a promising cofactor for antibiotic therapy in the treatment of joint prosthesis infections [66].

Sun H et al. (2021) presented the high potential of the usage of berberine-hybridised benzimidazoles as antibacterial agents in the treatment of animal *S. aureus* infections (MIC = 0.006 mmol). The bacterial cells and biofilm destruction were observed with minimal toxic effects on the body [69]. In another study, the antimicrobial activity of pure berberine was confirmed, as was its synergistic activity with clindamycin and rifamycin against MRSA. Furthermore, there was a 24 h reduction in the number of bacteria by 2 log CFU·mL^−1^ (CFU, colony-forming unit) and a decrease in the ability of the pathogen to form a biofilm. Berberine is attributed to the direct destruction of the cell wall structure and violation of the integrity of the bacterial cell membrane [70]. The study by Zhang X et al. (2020) confirmed that the cell wall of methicillin-resistant *S. aureus* can be affected in the same manner [71]. On the other hand, Huang X et al. (2020) presented a new concept for treating MRSA infections. The idea concerns the production of nanoparticles based on the combination of berberine and cinnamon acid (CA) and their ability to facilitate the intracellular penetration of multi-drug-resistant bacteria. The results showed that BRB-CA NPs decrease the pathogen’s ability to form a biofilm and, at the same time, do not cause any hemolytic effect on the body cells [72].

The combination of berberine and fusidic acid (FA) demonstrated excellent synergistic properties against MRSA. After incubation with BRB + FA for 24 h, a decrease in the count of MRSA strain 4806 (by 4.2 log CFU·mL^−1^) and biofilm formation (3.0 log CFU·mL^−1^) was observed [73]. In another study, berberine constricted the formation of *S. aureus* biofilm by inhibiting PSM (phenol soluble modulin) aggregation, which destabilised the biofilm amyloid structure and prevented its formation [74]. Fan et al. (2018) described the antimicrobial activity of cycloberberberine derivatives against MRSA and MSSA (methicillin-sensitive *S. aureus*). The MIC values ranged between 1 and 4 μg·mL^−1^ [75]. Additionally, simultaneous administration of BRB and curcumin in liposomal form improved the penetration capacity of these compounds into MRSA cells and reduced the MIC values of both substances (which indicate that both substances had synergistic effects). Another study revealed that berberine significantly reduced *S. aureus* biofilm formation along with the ability to infect host cells and inflammation severity [76]. An interesting report on the potential synergy of BRB with thymol (TH) in the treatment of *S.aureus* infections can be found in the article by Aksoy et al. (2020). A BRB-TH combination inhibited *S. aureus* biofilm formation and therefore could be considered a synergistic anti-virulent factor [77].

##### CoNS (Coagulase-Negative Staphylococcus)

Synergistic effects of berberine and antibiotics from the oxazolidinone, macrolides, and beta-lactam groups were observed in in vitro analysis. These findings suggest that BRB is responsible for blocking the NorA efflux pump. The MIC values varied between 16 and 512 µg·mL^−1^. This premise is of great importance due to the fact that CoNS (i.e., *Staphylococcus epidermidis*) are a frequent cause of severe wound infections, which can be fatal [78]. BRB was evaluated by Zhou X et al. (2015) as an agent that enhances the efficacy of conventional antibiotics against MDR *S. epidermidis*, which causes mastitis in cows. The results are promising because berberine acted synergistically with lincomycin, amoxicillin, and penicillin in the treatment of these infections (MIC ranged from 2 to 512 μg·mL^−1^) [79].

##### *Enterococcus* spp.

In the Chen L et al. (2016) study, the impact of berberine on the ability to create the biofilm by *Enterococcus faecalis*, isolated from patients with UTI (urinary tract infection), was assessed. The findings showed that BRB inhibited the formation of biofilm as a result of its impact on the expression of sortase genes (*srtA* and *esp*) [80].

##### *Klebsiella* *pneumoniae*

Several studies have documented the effect of berberine on *K. pneumoniae* strains. For example, the impact of six protoberberine alkaloids on MDR pathogens was evaluated in the study by Guefack et al. (2022). The observed MIC values were below 100 μg·mL^−1^ for BRB for *E. coli* (ATCC 10536, AG102) and *K. pneumoniae* (KP55, ATCC 11296, MRSA-3, MRSA-6). The antibacterial effects of the following antibiotics: norfloxacin, ciprofloxacin, and doxycycline, have been enhanced by some of the tested alkaloids, indicating synergistic effects [81]. Zhou et al. (2016) evaluated the in vitro activity of ciprofloxacin in combination with berberine against clinical isolates of *K. pneumoniae* (including ESβL-extended-spectrum beta-lactamases). The complex of BRB + CYP resulted in a reduction of MIC values for both substances at least by half. It was observed that the synergistic (in 4 out of 22 cases) and additive (in 17 out of 22 cases) effects of both substances Simultaneous application of BRB and CYP decreased the MIC value of quinolone, which led to an increase in the antibacterial activity of CYP against *K. pneumoniae* [82].

##### *Acinetobacter* *baumannii*

In vitro tests have shown that berberine exhibits synergistic effects with various drugs. Despite its low activity in monotherapy, BRB was able to restore susceptibility to antibiotics (e.g., tigecycline, meropenem, ciprofloxacin, and sulbactam) in multi-drug-resistant *A. baumannii*. It is assumed that berberine can be used clinically due to the sensitisation of MDR pathogens to several antibiotics to which they were originally resistant [83]. In the work of Gao W et al. (2018) on the effect of the 2-aminothiazole-berberine derivatives on MDR *A. baumannii* (MIC = 2 nmol·mL^−1^, low cytotoxicity), the analysed compounds were found to destabilise the cell membrane of bacteria as well as intercalate and cleave the DNA of the pathogen [84]. In the Ahmadi et al. (2022) study, the effectiveness of berberine, thioridazine (TRD), and ciprofloxacin in reducing the expression of the *adeABC* efflux pump in MDR *A. baumannii* was evaluated. The authors showed that thioridazine alone (MIC = 64 µg·mL^−1^) and the combination of CYP or BRB with TRD significantly reduced the expression of the *adeB* gene. The study is an important premise for further research on the use of BRB as a cofactor for the reduction of MIC values for quinolones and breaking the multi-drug resistance of Gram-negative pathogens [85].

##### *Escherichia* *coli*

In the study by Thakur et al. (2016), the synergistic effects of the aqueous extract of *Berberis aristata* (including berberine) with 3-line antibiotics (colistin, tigecycline, and amoxicillin-clavulanate) used in the treatment of carbapenem-resistant *E. coli* (NDM-1, the New Dehli metallo-β-lactamase 1) infections were demonstrated. For colistin and tigecycline, the MIC value reduction was practically 50%, making these combinations an interesting strategy for treating carbapenem-resistant *E. coli* infections [86]. The study by Bandyopadhyay et al. (2013) showed the potential therapeutic application of berberine in the treatment of infections in animals with MDR *E. coli* etiology (ETEC, Enterotoxigenic *E. coli*; EPEC, Enteropathogenic *E. coli*). The antibacterial effect of BRB in a dose-dependent manner, with MIC 50 against ETEC at 1.75–196 μg·mL^−1^ and 2.07–3.6 μg·mL^−1^ against EPEC, was observed. The test results are promising, but they should be confirmed in large-scale studies [87].

Vital reports on the use of berberine as an *Escherichia coli mdfA* efflux pump inhibitor were provided by Li Y et al. (2023). The authors showed that the sensitivity of *E. coli* to CYP has been restored by this active compound (20 µg·mL^−1^ of BRB, resulting in a decrease in the MIC value of CYP from 0.032 to 0.008 µg·mL^−1^). These findings are highly significant in view of the growing global resistance of bacteria to antibiotics [88]. It was also reported that MDR-*E. coli* can be affected by nitroimidazole BRB derivatives. One of these compounds (MIC = 0.003 mmol) showed antibacterial activity 33 times higher than norfloxacin, while at the same time not having significant cellular toxicity [89]. The noteworthy research on the activity of berberine-derived azolyl ethanol against MDR *E. coli* was conducted by Sun et al. (2021). It was shown that two derivatives were characterised by low systemic cytotoxicity and low MIC values (0.007 and 0.006 mmol) [90].

##### *Pseudomonas* *aeruginosa*

The use of berberine against aminoglycoside-resistant *P. aeruginosa* (via the MexXY-OprM efflux pump) has been described by Morita et al. (2016). For instance, the study showed a decrease in resistance to amikacin after the in vitro use of BRB. Furthermore, berberine has been shown to restore susceptibility to aminoglycosides in *Burkholderia cepacia* and *Acintetobacter xylosoxidans*. Importantly, the blockage of the MexXY-OprM efflux pump by BRB also restored the sensitivity of *P. aeruginosa* to cefepime, erythromycin, and lincomycin. Moreover, berberine intensified the synergistic effect of amikacin with piperacillin against MDR strains of *P. aeruginosa* [91]. The synergy of azithromycin combination (AZM) with BRB against *P. aeruginosa* clinical isolates from patients with cystic fibrosis was demonstrated in in vitro and in vivo studies. The use of the combination BRB-AZM decreased the formation of key virulence factors by bacteria and reduced biofilm formation [92]. Similar findings on the synergistic activity of BRB and AZM in the reduction of MIC values are presented in the Zhao Z et al. (2022) study. The authors emphasised their effectiveness in inhibiting *P. aeruginosa* growth, along with a significant decrease in the production of alginate biofilm by the pathogen [93].

Numerous studies have confirmed the beneficial effects of berberine against *P. aeruginosa* strains that are resistant to aminoglycosides. This natural compound is able to inhibit the MexXY-OprM efflux pump, thus reducing the classic *P. aeruginosa* resistance mechanism based on the efflux removal of the antibiotic molecule outside the cell. The authors demonstrated the effectiveness of the combination of aminoglycoside (tobramycin) with BRB in in vitro tests against clinical isolates of *P. aeruginosa*. Higher levels of bactericidal activity of aminoglycosides were obtained, which highlights the potential of combining with BRB in restoring the sensitivity of *P. aeruginosa* to these drugs [94,95,96]. In another study, di-berberine was investigated as a *P. aeruginosa* efflux pump MexXY-OprM inhibitor. Similarly to BRB, di-berberine is able to act with aminoglycoside antibiotics in a synergistic manner to overcome bacterial resistance [97]. In the study by Su F et al. (2017), the synergistic effect of BRB on imipenem (IMI) against *P. aeruginosa* (PA012, a carbapenem-resistant strain) was confirmed. The combination of BRB (1/4 MIC) with IMI (1/8 MIC) triggered a synergistic effect between these substances. Due to better IMI activity, BRB could more efficiently penetrate bacterial cells (Su F. et al. 2017). The in vitro evaluation of the effects of BRB and palmatine on the restoration of CYP sensitivity in MDR *P. aeruginosa* isolated from burn wounds was conducted by Aghayan et al. (2017). Both substances were characterised as efflux pump inhibitors. A decrease in the MIC value of CYP was detected in 91.67% of strains in therapy combined with BRB [98].

#### 2.1.2. Biological Activity

The molecular activity of berberine was evaluated on the basis of the model strain *S. aureus* (ATCC 25923) [99]. The authors used metabolomic tools to indicate the influence of BRB metabolites on the vital functions of bacterial cells. It was found that berberine induced a global inhibition of the metabolism of cellular nucleic acids and amino sugars. Reduced stress resistance and induced generation of phospholipid peroxides with a simultaneous decrease in the creation of antioxidants (e.g., γ-tocopherol and farnesyl-PPi) were also observed. The process was accompanied by the accumulation of cellular wall precursors (UDP-MurNAc, UDP-Glic-NAc, and UDP-Man-NAc) and inhibition of D-Ala-D-Ala synthesis. Most likely, these results can be attributed to the blockage of the synthesis of peptidoglycan in bacterial cells. The inhibition of spreading of *S. aureus* biofilm was associated with a decrease in the expression of the *agrA* (QC-associated gene) caused by the sub-inhibitory concentration of BRB [64].

There is also evidence of the synergistic effects of berberine with tocarol against planktonic and biofilm forms of *S. aureus*. Molecular analysis of PCR showed that under the influence of the substance, there was a reduction in the expression of the *sarA*, *cidA*, and *icaA* genes involved in the formation of the *S. aureus* biofilm [100]. Another molecular study suggests that the blocking effect of cycloberberine derivatives on the FtsZ protein may induce the inhibition of cell division in MRSA [75]. Berberine also induced the expression of key virulence factors in *K. pneumoniae*. The acrAB-TolC efflux pump was overexpressed by BRB, which led to a negative impact on bacterial cell growth at the molecular level [82].

The multi-omics study on the berberine mechanism of action in *E. coli* cells revealed that it can trigger nucleic acid metabolism dysfunction, cellular wall damage, and the inhibition of transmembrane transport [101]. Berberine can also inhibit the expression of crucial *Klebsiella* spp. genes involved in biofilm formation, cell division, and quorum sensing (*luxS*, *pfS*, *hflX*, *ftsQ*, *uxS*, and *ftsE*) [102]. Zhang et al. (2018) proved that the antibacterial effect of berberine is probably associated with its ability to disrupt bacterial DNA synthesis by producing DNA-BRB derivative complexes [89]. The berberine-derived azolyl ethanol was characterised by its ability to inhibit the formation of *E. coli* biofilm by decreasing the formation of exopolysaccharides by bacteria. The mechanism of their molecular action was based on bacterial DNA intercalation, cell membrane degradation, and increasing cell oxidative stress [90].

It is worth noting that berberine owes its effect to the inhibition of the MexXY-OprM efflux pump against *P. aeruginosa* [91,94,95,96,103]. BRB and aztreonam combination resulted in a reduction in the formation of important virulence factors by *Pseudomonas* spp., i.e., LasA, LasB, alginate, chitinase, pyocyanin, pyoverdine, etc. Biofilm creation has been weakened as a result of a decrease in quorum sensing activity. In vivo, a reduction of inflammation in the lung area and an increased survival of experimental mice were observed [92]. Demethyleneberberine was indicated as a possible therapy for pneumonia caused by *P. aeruginosa*. This research suggests that berberine can be responsible for the induction of AIM2 inflammasome inhibition, which ultimately led to a decrease in inflammation in the murine model of infection. The formation of the pro-inflammatory cytokine IL-1β and oxidative stress have been lowered [104]. Aswathanarayan et al. (2018) demonstrated that berberine has an inhibitory effect on the formation and maturation of *P. aeruginosa* (PA01) biofilm formation. Bacteria have also shown a significant decrease in biomass growth and cell mobility, as well as an inhibition of QS signalling [105]. The combination of BRB and tigecycline caused a reduction in the expression of crucial pump genes like *mexX*, *mexY*, *mexZ*, and *oprM* (which encodes the MexXY-OprM efflux pump components) [103].

### 2.2. Catechin (CT)

**Figure 3:**
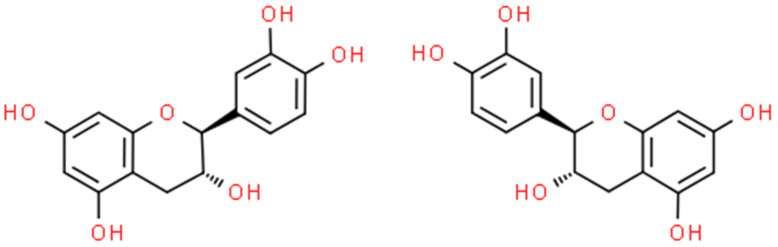
L-(–)-catechin (**on the left**) and D-(+)-catechin (**on the right**) structures (ChemSpider database).

**Figure 4:**
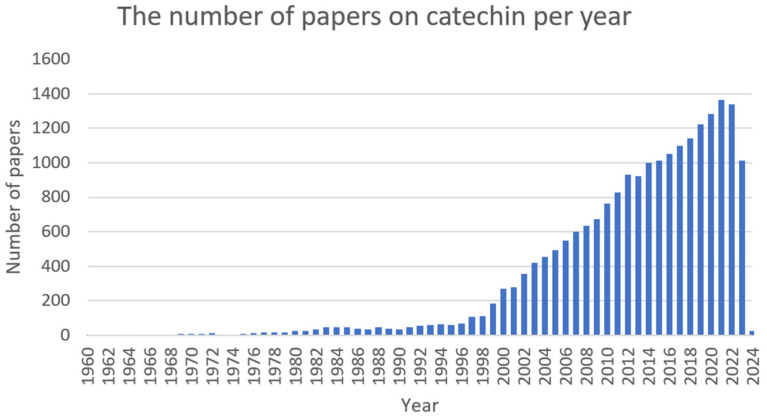
The number of papers on catechin per year (PubMed database).

Catechins (Figure 3) are natural polyphenolic compounds—flavan-3-ols (or flavanols); which belong to the flavonoid family [106,107]. They can be classified into two groups: free and esterified catechins [107]. The group of non-esterified catechins includes galocatechin, epicatechin, and epigallocatechins, while the group of esterified catechins comprises epigallocatechin gallate, epicatechin gallate, gallocatechin gallate, and catechin gallate [107]. These compounds are present at high concentrations in various fruits, vegetables, and beverages, for example: grapes (*Vitis* L.), blackberries (*Rubus* L.), raspberries (*Rubus* L.), apples (*Malus* Mill.), pears (*Pyrus* L.), cherries (*Cerasus* Mill.), green tea (*Camellia sinensis*), chocolate, and legumes [108]. Notwithstanding, most studies focus on teas derived from *Camellia sinensis* when assessing the amount and activity of catechins [109,110]. The amount of catechin in these natural sources is dependent on several factors, among others: variety, location and cultivation practices, season, illumination, and altitude [107]. Despite the fact that these compounds are not crucial in human nutrition, they can support well-being by preventing different ailments [108]. Numerous studies have shown the varying biological activities of these compounds, i.e., antibacterial [111], antifungal [112], antiviral [113], anticancer [114], antimutagenic [115], antiproliferative [116], antioxidative [117], anti-inflammatory [118], UV protection [119], and anti-neurodegenerative [120]. The number of articles on catechin is shown in Figure 4.

#### 2.2.1. Antimicrobial Activity

##### *Staphylococccus* *aureus*

Catechin was studied by Veiko et al. (2023) to assess its effect on the hemolytic activity of *S. aureus* (NCTC 5655) induced by α-hemolysin. No significant effect of this bioactive compound on enzyme activity or destabilisation of the bacterial cell membrane was observed (which correlates with its MIC value = 150 μmol) [121]. In the study by Miklasiska et al. (2016), the antibacterial activity of CT and its interaction with classic antibiotics were assessed. Catechin showed MIC values at 256–2048 μg·mL^−1^ against *S. aureus* isolates, including MRSA and MLSB (resistance to macrolide, lincosamide, and streptogramin B). Synergistic effects with clindamycin and erythromycin have been demonstrated. It was noted that catechin can effectively reduce the MIC values of antibiotics against MRSA pathogens. However, such a beneficial effect was not observed for MLSB *S. aureus* [122].

Another study determined the activity of (+)-catechin, (−)-epigallocatechin, and (−)-epicatechin gallate against MRSA and their effect on the activity of β-lactam antibiotics. The in vivo murine sepsis model demonstrated the effectiveness of CT and epicatechin combination with oxacillin as the most effective in reducing the number of bacteria in the blood of MRSA-infected mice. The complex of CT and epicatechin intensified the action of β-lactam antibiotics (oxacillin, ampicillin, ampicillin/sulbactam, cefazoline, cefepime, and imipenem/cilastatin) against MRSA. The detection with daunorubicin showed an increase in the cellular accumulation of the antibiotic when co-administered with CT and epicatechin. At the molecular level, the bacterial gene expression decreased for *norA*, *norC*, and *abcA* (MRSA efflux pump genes) [123]. Another survey comprehensively assessed the effect of various flavonoids, among them catechin, on pathogens present in plaque, including *S. aureus*, *E. coli*, and *E. faecalis*. The CT did not show activity against *E. coli* and *E. faecalis*, while the MIC value for *S. aureus* amounted to 3550 μmol (1000 μg·mL^−1^) [124]. The efficacy of catechin 3-O-ramnoside against several pathogens, including MRSA, *E. coli*, and *K. pneumoniae*, has also been demonstrated. The compound’s MIC values extended from 6.25 to 12.5 μg·mL^−1^. The CT derivative was not active against VRE (vancomycin-resistant *Enterococcus*) [125].

##### *Staphylococcus* *epidermidis*

The effect of poly-CT on *S. epidermidis* adhesion to silicone and polyurethane urological catheters was examined. The use of catechin polymer led to a significant reduction of *S. epidermidis* and *E. coli* biofilm adhesion on the silicone catheter and polyurethane catheter by 81% and 96%, respectively [126].

##### *Enterococcus* spp.

The effect of CT (an extract of *Uncaria gambir*) on the potential inhibition of *E. faecalis* QS by blocking GBAP protein, gelatinase, and serine proteases was investigated. The MIC and MBC values of catechin amounted to 0.625 μg·mL^−1^ and 1.25 μg·mL^−1^, respectively. This study confirmed a higher activity of CT than fosfomycin against the tested bacteria strain (ATCC 29212). The in silico study revealed a significant CT binding affinity between MurA, GBAP, and gelatinase. According to this study, catechins exhibit the ability to inhibit QS *E. faecalis*, which could prevent the formation of bacterial biofilm [127].

##### *Klebsiella* *pneumoniae*

The use and effectiveness of catechins against *K. pneumoniae* are not well documented. There are reports on the antimicrobial activity of extracts (containing a flavonoids mixture) obtained from *Hedera helix* (gallic acid and CT) [128] and *Teucrium* (including CT) [129], which obtained similar results—slight activity against *K. pneumoniae*. In the work of Alqahtani et al. (2022), the synergistic effect of CT (extract from *Ficus retusa*) with tetracycline against *E. coli*, *P. aeruginosa*, and *S. aureus* and the additive against *K. pneumoniae* (the MIC value for CT = 2.33 mg·mL^−1^) was presented [130]. In an in vivo model of rat skin infection, the antimicrobial activity of extracts from the fruit of *Parkia clappertoniana* (containing quercetin and CT) and their effects on wound healing were investigated. The results showed that the obtained extracts exhibit a comparable cyprofloxacin growth inhibitory capacity (in a concentration-dependent manner). At a concentration of 4 MIC, the bacteriostatic effects were achieved against *Klebsiella* spp. and lasted for 24 h [131]. Another study showed that *Punica pomegranate* extract (containing 151 mg·g^−1^ of CT) had an antibacterial effect against *K. pneumoniae* (ATCC 13883) (MIC = 160 μg·mL^−1^) [132].

##### *Acinetobacter* *baumannii*

Ibitoye et al. (2019) studied the effects of (+)-catechin on *A. baumannii* strains (AB5075, ΔsodB, ΔkatG) and the antimicrobial activity of quinolone antibiotics (CYP) and gemifloxacin (GEM). The MIC value of 256 μg·mL^−1^ for strain AB075 was observed. For mutants, in terms of dismutase and catalase genes, significantly reduced MIC values (64 and 128 μg·mL^−1^) were noted. This can be a confirmation of the severity of cell oxidative stress. The synergism of the combination of CYP and GEM based on the intensification of ROS (reactive oxygen species) synthesis by quinolones has been demonstrated [133]. It has also been shown that blue light and catechin can result in the photo-induced formation of ROS. The increased oxidative stress conditions can effectively eliminate *A. baumannii* (71.8% inactivation of the pathogen at 145 μg·mL^−1^ concentration of CT concentration and 60 min exposure), including carbapenem-resistant strains (CRAB, Carbapenem-resistant *A. baumannii*, inactivation of 93.8% at 290 μg·mL^−1^ concentration of CT and 2 h exposure [134].

Another article revealed the catechin docking capabilities in relation to the BfmR protein responsible for controlling the formation of biofilm in *A. baumannii*. The MIC values of CT against *A. baumannii* A75, A76, and A77 strains (all formed an efflux multi-drug resistance pump; strain A77 was also a MβL producer) were 25, 12.5, and 12.5 μg·mL^−1^, respectively. The catechin also reduced the biofilm formation by approximately 70% against the A75 and A76 strains and by 47% against the A77 strain [135]. The effects of *Boletus edulis* and *Neoboletus luridiformis* extracts containing catechin against clinical ESKAPE isolates from wounds (*E. faecium*, *S. aureus*, *K. pneumoniae*, *A. baumannii*, *P. aeruginosa*, and *Enterobacter* spp.) were also examined. The extracts tested were able to limit the growth of *S. aureus* and *E. coli* and also inhibit the formation of their biofilms. However, no significant activity against *A. baumannii* was demonstrated [136].

##### *Pseudomonas* *aeruginosa*

In Abdel et al. (2022), the antimicrobial activity of *Pelargonium* extract containing catechin and gallic acid was evaluated against clinical isolates of *P. aeruginosa*. As a result of their application, the percentage of pathogens forming biofilm decreased from over 50% to 5.26%. The survival parameters for infected animals (murine in vivo model) were higher among individuals exposed to catechin with gallic acid [137]. The effect of silver nanoparticles with catechin on the healing process of *P. aeruginosa*-infected wounds in an in vitro and in vivo model, including the antibacterial activity of compounds, was evaluated by Kalirajan et al. (2020). The study showed the formation of a growth inhibition zone (2.9 cm for 2% CT and 3.4 cm for 5% CT) around the compound complex. In addition, after the incubation of substances with *P. aeruginosa*, the growth of pathogens in the control culture has not been observed. The in vivo model showed accelerated healing activity after the addition of catechin (100% after 24 h) to the *Pseudomonas* spp.-infected burn wound [138]. The study assessed the effect of rare earth ions (lanthanum, ytterbium, and gadolinium) nanoparticles combined with catechin on the adhesive capacity and the formation of *P. aeruginosa* biofilm. The great antibiofilm properties and significant inhibition of pathogen growth have been demonstrated [139].

The potential synergistic properties of catechin with classic antibacterial agents against *P. aeruginosa* (including MDR strains), *E. coli*, and *S. aureus* were also assessed. The synergistic effects of CT on norfloxacin and gentamicin and the antagonistic effects of tetracycline were detected in *S. aureus*. Furthermore, the antagonistic effect of erythromycin was confirmed in *P. aeruginosa*. In addition, CT showed synergistic activity with imipenem and tetracycline in *E. coli* strains [111]. The oxidative stress conditions in pathogenic bacterial cells increased under the influence of (+)-catechin. The MIC and MBC values against *P. aeruginosa* were 600 and 800 μg·mL^−1^, respectively [140].

##### *Escherichia* *coli*

In another study, catechin, catechic acid, and vanillic acid were examined as potential solutions for the inhibition of the growth and creation of biofilm in *E. coli* (a UPEC, uropathogenic *Escherichia coli* strain). The MIC value for pure catechin was set at 13.78 mmol, and the MBC value was not specified. The active compounds showed MIC values against planktonic forms of pathogens when mixed at the following concentrations: 0.05 mmol, 1.62 mmol, and 0.74 mmol, respectively. In the case of the eradication of the biofilm, the combination at concentrations of 1.72 mmol, 3.2 mmol, and 2.97 mmol, respectively, proved to be the most effective. The mixture of these three active compounds was more efficient at eliminating previously formed biofilm on the silicone surface than nitrofurantoin. The catechin demonstrated an advantageous profile in the reduction of biofilm biomass [141]. The impact of catechin, catechic acid, and vanillic acid on the inhibition of *E. coli* (UPEC) adhesion on the surface of a silicone urological catheter was also assessed by Bernal-Mercado et al. (2020). The authors presented that sub-inhibitory concentrations can reduce the adhesive capacity after 8 h of incubation (a decrease of 2.03 log CFU·cm^−2^). It was also noted that the active compounds were more effective in combination than separately [142].

Diaz-Gomez et al. (2014) revealed the effects of catechin and gallic acid on *E. coli* and investigated the interactions between both substances. The CT at a concentration of 5 mg·mL^−1^ showed poor inhibitory capacity for bacterial growth in comparison to gallic acid. A reduction in the number of pathogens from 6·10^8^ CFU·mL^−1^ to 4·10^8^ CFU·mL^−1^ was observed. Furthermore, no synergistic relationship between these compounds was demonstrated. The study proved the superiority of gallic acid over catechin in its antibacterial activity [143]. Another survey demonstrated that catechin and epigallocatechin gallate present in *Camellia sinensis* tea extract can reduce the severity of colitis caused by pathogenic *E. coli* strains (F18: LT: STa: Stx2e) (the MIC value = 40 mg·mL^−1^). The in vivo model showed a decrease in bacterial cell count. After 10 days of treatment, there was a decrease in the CFU value by 94% (at a concentration > 60 mg·mL^−1^) [144].

#### 2.2.2. Biological Activity

There was a decrease in the expression of the genes (*lasR* and *lasI*) responsible for quorum sensing virulence factors among *P. aeruginosa* after exposure to catechin. The in silico model exhibited that CT in combination with gallic acid can directly block the LasR, which makes them a promising QS inhibitor in *Pseudomonas* spp. [137]. The exposure to CT induced the expression of peroxide dismutase and catalase in bacterial cells and increased ROS generation. The reduced glutathione concentration and increased amount of DNA broken particles inside the cells provide strong evidence of the growing oxidative stress [140].

### 2.3. Chelerythrine (CHE)

**Figure 5:**
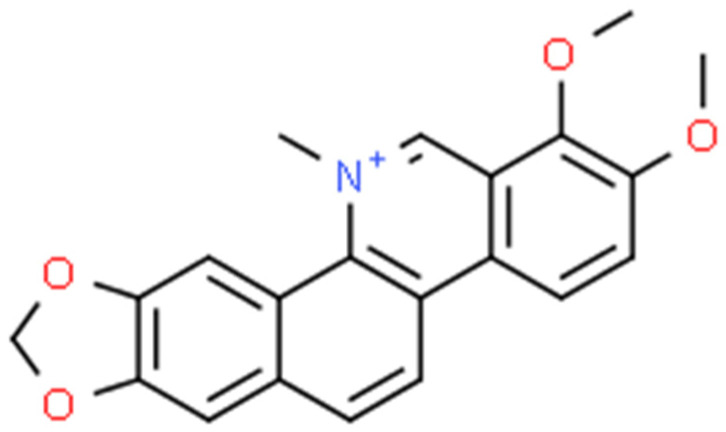
Chelerythrine structure (ChemSpider database).

**Figure 6:**
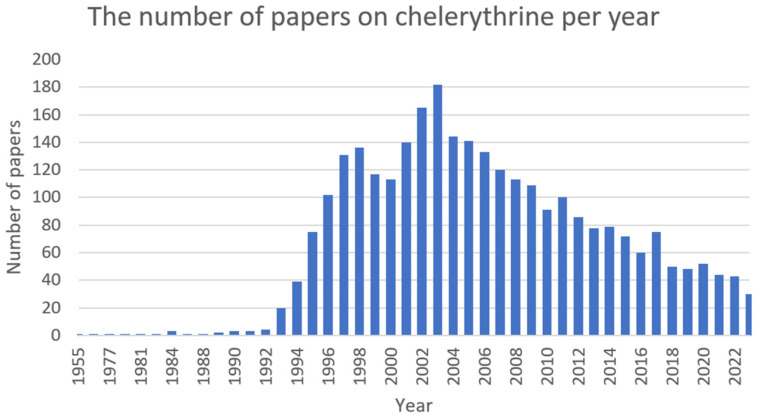
The number of papers on chelerythrine per year (PubMed database).

Chelerythrine (Figure 5) is a type III benzophenanthridine alkaloid isolated from four plants of the families *Papaveraceae* (e.g., *Chelidonium majus* L., *Macleaya cordata* (Willd.) R.Br., and *Sanguinaria canadensis* L.) and *Rutaceae* (e.g., *Zanthoxylum asiaticum* (L.) Appelhans, Groppo & J.Wen) [145,146,147]. This bioactive compound possesses anti-inflammatory [71], antibacterial [148], antifungal [149], and antitumor [150] activities. The increase in interest among scientists is clearly visible in Figure 6.

#### 2.3.1. Antimicrobial Activity

##### *Staphylococcus* spp.

In the study carried out by Qian et al. (2020), the effect of chelerythrine on a dual-species biofilm formed by two bacteria, *S. aureus* (ATCC 25923) and *Staphylococcus lugdunensis* (ATCC 700328), was assessed. For the MIC value of 8 µg·mL^−1^, the effect of CHE was recognised as antimicrobial. However, a concentration of 4 µg·mL^−1^ affects the initial stage of adhesion (i.e., biofilm formation). At a concentration of 256 µg·mL^−1^, chelerythrine has the ability to reduce mature biofilm by 90% within 24 h. Studies performed using CVSA (biofilm analysis by crystal violet staining), ESEM (environmental scanning electron microscopy), and CLSM (laser scanning microscope) showed a significant decrease in the amount of biofilm at the MIC value of 2 µg·mL^−1^. Furthermore, CLSM displayed damage to the membrane of cells surrounded by dual-species biofilms with a dose-dependent effect (a higher dose of 4 MIC gave a better damaging effect than the dose of 2 MIC). CHE enhances the killing effect of antibiotics by promoting increased biofilm permeability [151].

##### *Escherichia* *coli*

The activity of chelerythrine against *E. coli* strains has also been proven. It was isolated from the *Chelidonium majus* plant, and its antibacterial activity against *E. coli* and *Bacillus subtilis* was tested using thin-layer chromatography combined with direct bioautography (TLC-DB). The test results showed its activity (light zone in the bioautogram) against both microorganisms, indicating better activity against *B. subtilis* [152].

In the research by Tavares et al. (2014), chelerythrine was obtained from the *Zanthoxylum rhoifolium* plant, which has a minimum inhibitory concentration of 1.50 µg·mL^−1^ for all bacteria tested in the study. It was chosen to represent a compound that has anticancer, anti-inflammatory, and antimicrobial properties. It was demethylated to obtain a compound with less activity than chelerythrine. It also has stronger antimicrobial properties than other alkaloids isolated from the tested plant. Additionally, a protective effect on erythrocytes was found, as indicated by a reduction in the values of the AOPP (advanced oxidative protein products) and TBARS (formation of substances reactive with thiobarbituric acid) tests, which indicates a quantitative measurement of lipid peroxidation in erythrocytes [153].

##### *Pseudomonas* *aeruginosa*

Chelyrethrine obtained from the root of *Chelidonium majas*, which contained a larger amount of alkaloids, was used in this study to check the MIC value, which for *P. aeruginosa* was 1.9 mg·L^−1^. At a concentration of 3.9 mg·L^−1^, chelerythrine did not show any cytotoxic effect. In combination with chelidonine and sanguinarine at a concentration of 6.25 mg·L^−1^, the mixture inhibited nearly 100% of the bacterial inoculum, indicating the good effect of these compounds in combination [154].

##### *Serratia* *marcescens*

The potential for using chelerythrine in alternative therapy was confirmed by Qian et al. (2021). The authors demonstrated the inhibitory effect of this compound (at sub-MIC concentrations) on biofilm formation by carbapenem-resistant strains of *Serratia marcescens*. They observed a very strong effect of chelerythrine on the production of biofilm components, resulting in damage and even the removal of biofilm-forming cells [155].

#### 2.3.2. Biological Activity

Chelerythrine is also present in the roots of *Toddalia asiatica*. Studies conducted by He et al. (2018) demonstrated the effect of this active compound on the wall of *S. aureus* (25.923, MRSA, and ESBLs-SA) through increased leakage of alkaline phosphatase outside the bacterial cell, suggesting the destruction of the bacterial cell wall. Changes occurring in the microbial cell membrane were also assessed. Membrane permeability increased, resulting in increased leakage of bacterial proteins within 2.5 h. Then, this phenomenon began to subside, which could be due to a decrease in the activity of chelerythrine or the initiated auto-healing mechanism of the bacteria [148].

### 2.4. Cinnamaldehyde (CAN)

**Figure 7:**
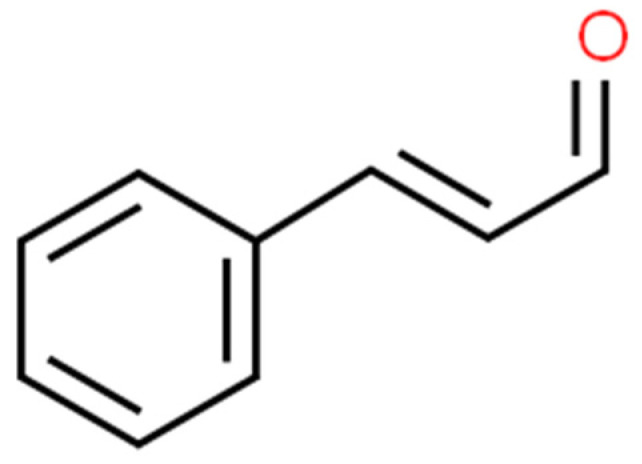
Cinnamaldehyde structure (ChemSpider database).

**Figure 8:**
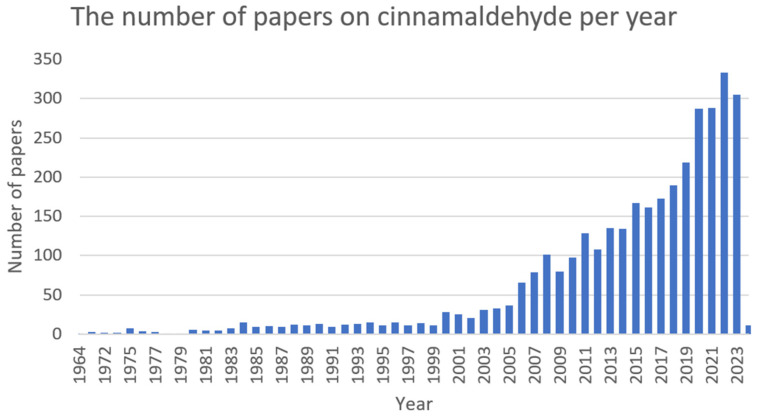
The number of papers on cinnamaldehyde per year (PubMed database).

Cinnamaldehyde (Figure 7) is a bioactive part of cinnamon essential oil and can be extracted from bark, leaves, and twigs of the genus *Cinnamomum*, which consists of several hundred species [156,157,158,159]. It possesses noticeable health benefits and various applications in the development of pharmacotherapeutic and nutraceutical foods [159]. Cinnamaldehyde has well-documented (Figure 8) activities, including: antimalarial [160], anticancer [161], antifungal [162], antiviral [163], antibacterial [164], anti-diabetes [165], antioxidative, antiperoxidative [166], and anti-inflammation [167].

#### 2.4.1. Antimicrobial Activity

##### *Staphylococcus* *aureus*

In the Zhang et al. (2014) study, the inhibitory effect of cinnamaldehyde and other substances of natural origin on the formation of mixed biofilms of *S. aureus* and *Salmonella enterica* subsp. *enterica* serovar Enteritidis was assessed. Bacteria’s biofilm formation was significantly inhibited by CNA, not by blocking QS but through the increased expression of AI-2, which is the bacterial communication signal autoinducer [168]. Cinnamaldehyde increases the permeability of the bacterial cell membrane, which induces its bactericidal effect. Activity against the Gram-negative (*E. coli*) and Gram-positive (*S. aureus*) bacteria was described in the study by Shen S et al. (2015). The MIC value for the activity of CNA amounted to 0.31 mg·mL^−1^ [169]. Moreover, CNA also has the potential to inhibit the virulence factors of multi-drug-resistant pathogens such as *S. aureus* and *E. faecalis*. The ability of bacteria to cause hemolysis of blood cells has been weakened by CNA, and cell adhesion to latex has also decreased. Importantly, CNA impact resulted in the cell wall lysis of *S. aureus* in a concentration-dependent manner. The in vivo study on the *G. melonella* larva model showed that CNA has the potential to increase the survival rate of individuals while limiting the number of bacteria in their hemolymph [170].

In addition, cinnamaldehyde was examined in another study to determine its impact on *S. aureus* in a murine wound infection model. The study group administered CNA in the amount of 200 μg per wound per day for 10 days after infecting a skin wound. Based on histological evaluation, it was claimed that CNA treatment relieves the severity of inflammation, accelerates wound healing, and also reduces the bacterial population in the wound. The results validated the efficacy of CNA as a potential preparation for the treatment of wound infections [171]. The effect of trans-CNA on antibiotics commonly used in infections caused by MRSA was also evaluated. The CNA has been shown to reduce *S. aureus* MDR resistance to amikacin, gentamicin, vancomycin, and amoxicillin by inducing a significant synergistic effect [172]. The results of the study conducted by Hu Z. et al. (2023) also showed the destructive effect of CNA on the cell wall and a reduction in MRSAs ability to form biofilm. Furthermore, the ability to degrade cell walls was increased when CNA was combined with ultrasounds (US). The simultaneous usage of US and CNA resulted in synergistic action due to the easier penetration of CNA through the cell membranes damaged by US [173].

The interaction of nisin and cinnamaldehyde as a potentially usable combination against *S. aureus* (ATCC 29213) was investigated in in vitro tests by Shi C. et al. (2017). The activity of these two compounds was considered synergistic due to the enhanced parameters of membrane and cell wall destruction with their simultaneous usage [174]. In the study by Gomes et al. (2018), the potential inhibitory effect of the CNA chalcone derivative on the MRSA efflux pump was evaluated. The MIC value > 1024 μg·mL^−1^ did not provide any significant potential uses as a direct antibacterial agent. However, the ability to competitively block an efflux pump allows it to be used in combination with antibiotics to which MRSA is originally resistant [111]. A new study by Kim Y et al. (2022) has revealed that CNA and trans-CNA derivatives have a negative effect on the biofilms of *S. aureus* and *E. coli* (UPEC). The activity was demonstrated by the aldehyde nitro derivative (the MIC value = 100 μg·mL^−1^). The growth of *S. aureus* was significantly reduced, and the formation of biofilm was also inhibited [175]. The effectiveness of chitosan and cinnamaldehyde nanoparticles in inhibiting *S. aureus* biofilm formation has decreased by 61% due to their synergic activity and effective penetration into the biofilm. The MIC value of cinnamaldehyde was established at 1.25 mg·mL^−1^. Moreover, under the influence of these substances, bacterial cell wall lysis and an increase in cell membrane permeability were observed [176].

##### *Staphylococcus* *epidermidis*

Albano et al. (2019) conducted an in vitro study on the antibacterial activity of cinnamaldehyde and its effect on inhibiting *S. epidermidis* biofilm formation. The MIC values ranged from 300 to 500 μg·mL^−1^. Notably, the ability to kill the planktonic form and the biofilm-formation cells has been confirmed. Furthermore, it has been proven that a sub-inhibitory concentration of CAN can inhibit the formation of *S. epidermidis* biofilm. Cinnamaldehyde may have a synergistic effect with linezolid, which is crucial in view of the growing bacterial resistance to antibiotics [177]. The effective elimination of both planktonic forms and the pathogen-created biofilm of *S. aureus* by CAN, thymol, eugenol, and thymoquinone was confirmed. The synergistic effects of CNA and thymol, as well as their ability to inhibit the formation of biofilm and increase the permeability of the bacterial cell membrane, were reported [178].

##### *Enterococcus* spp.

Ali et al. (2021) showed that the sub-inhibitory concentration (3.7 mmol) of cinnamaldehyde can inhibit the growth of planktonic *E. faecalis* cells as well as the viability of the biofilm form. The decrease in biofilm formation is caused by the reduction of bacterial exopolysaccharide formation. The cinnamaldehyde also induced a decrease in *E. faecalis* gelatinase and cytolysin activity [179]. Akshaya et al. (2023) examined the effect of CNA on the formation of *E. faecalis* biofilm, the expression of pathogen virulence factors, and the activity of macrophages on their formation of nitrogen monoxide (NO) in an in vitro model. Cinnamaldehyde has been proven to inhibit ESP (*Enerococcal* Surface Protein), which is responsible for the formation of biofilm structures. However, statistically significant antimicrobial effects were obtained at concentrations above 1000 μmol. Furthermore, research has revealed that CNA also enhances NO formation and macrophage migration and improves their phagocytotic ability against bacteria [180]. In the work of Ali et al. (2020), an assessment of the effectiveness of trans-CNA in comparison to conventional root canal disinfection agents and the *E. faecalis* biofilm growth ability was conducted. The study showed that CNA had a noninferior inhibitory impact on the reduction of the viability of *E. faecalis* biofilm compared with sodium hypochlorite (1%) or chlorhexidine (2%) in the first 15 minutes after the beginning of the treatment. Additionally, the cinnamaldehyde has retained its properties on the biofilm longer than chlorhexidine (failure of therapy after 10 days) [181]. The Hu et al. (2022) study was conducted to investigate the interaction between CNA and sophorolipid acid to enhance the penetration capacity of *E. faecalis* biofilm. The observed ability to inhibit bacterial growth and the formation of biofilm was the highest for CNA in comparison to other compounds. Finally, the tested combination resulted in a higher ability to kill bacteria in the biofilm area as well as a reduction in biomass formation and biofilm volume [182].

##### *Klebsiella* *pneumoniae*

It has been demonstrated that cinnamaldehyde has strong antibacterial activity against both MDR *K. pneumoniae* strains resistant to carbapenems (KPC-2) and polymyxins (KPC-2 and *mgrB* gene mutants). A decrease in the total number of bacteria in the blood and serum fluids in the in vivo model after 24 h of exposure to CNA was observed [183]. Another study reported the effects of pure CNA alone and in combination with ciprofloxacin and cefotaxime against clinical isolates of *Enterobacteriaceae* ESβL and strains resistant to quinolones. The MIC value of CNA for *K. pneumoniae* was observed at 0.91 μg·mL^−1^. Moreover, the synergistic effects of cinnamaldehyde with cefotaxime (60.6%) and ciprofloxacin (42.4%) were noted [184].

##### *Acinetobacter* *baumannii*

In the Mohamed et al. (2018) study, the strong antibacterial and antibiofilm activity of cinnamaldehyde against carbapenem-resistant *A. baumannii* (CRAB) was demonstrated. The MIC value reached a concentration of 0.875 mg·mL^−1^, while the MBC value was observed at 1.75 mg·mL^−1^, against the planktonic form of bacteria. All 23 tested clinical isolates were characterised by their resistance to carbapenems; some of them were also resistant to other β-lactams, aminoglycosides, and quinolines, and four of them had a high ability to form a biofilm. The inhibitory rate of biofilm growth was 49.5% to 71.2% at 1/2 MIC. The results demonstrated a high level of antibacterial and antibiofilm activity and are a promising agent for the treatment of infections with CRAB etiology [185]. Another study showed the strong antibacterial effect of cinnamaldehyde against MDR clinical isolates of *A. baumannii* (the MIC values = 0.01–0.04%). Moreover, an additive activity of CNA and commonly used drugs (e.g., colistin, imipenem, ceftazidime, amikacin, and gentamicin) has been shown. The synergistic effect of colistin with CNA (against strains BF05, SF17, and PK06) and imipenem (against the *A. baumannii* SC29 strain) has been confirmed. The exposure of 2 MIC of CNA resulted in a molecular effect on cell division disorder, leading to the formation of elongated forms with abnormal morphology [158]. The bromine cinnamaldehyde derivatives have shown antibacterial activity against *A. baumannii* (the MIC value = 32 μg·mL^−1^), while the cinnamaldehyde derivative exhibited low systemic and hemolytic cytotoxicity to the model organism *Caenorhabditis elegans* (the MIC value = 64 μg·mL^−1^) in in vivo studies. It is crucial to note that the XDR (extremely drug-resistant) *A. baumannii* strain used in this study remained sensitive to the tested derivatives, and the sensitivity to these compounds was increased when an efflux pump inhibitor was added [186].

Trans-cinnamaldehyde and eugenol have been tested as potential substances that could restore sensitivity to β-lactam antibiotics (e.g., ampicillin, methicillin, meropenem, penicillin, aztreonam, piperacillin, and amoxicillin) in clinical isolates of *A. baumannii*. The simultaneous use of these active compounds caused a decrease in AdeABC efflux pump activity, which resulted in increased *A. baumannii* sensitivity to all tested antibiotics [187]. According to the in vitro study by Karumathil et al. (2016) on the wound infection model, eugenol and CNA inhibited the adhesion and invasion of human keratinocytes by clinical isolates of *A. baumannii*. Furthermore, these substances have the capacity to damage the biofilm and kill the cells that make up its structure [188].

##### *Pseudomonas* *aeruginosa*

In the Topa et al. (2020) study, the inhibitory activity of cinnamaldehyde on the development of biofilm and QS of *P. aeruginosa* as well as numerous interactions with classic anti-pseudomonas antibiotics were identified. The combination of CNA and colistin resulted in a decrease in its MIC value for antibiotics from 6.8 μmol to 1.7 μmol, which was defined as a synergistic effect. The mixture of CNA with colistin or tobramycin led to increased inhibition of biofilm formation by the pathogen by 83.9% and 75.2%, respectively (for pure drugs, it was 35%) [189]. Another study demonstrated that trans-cinnamaldehyde inhibited *P. aeruginosa* biofilm formation (the MIC value = 0.8 mg·mL^−1^) and led to its destruction. During 48 h of exposure to CNA at a sub-inhibitory concentration (0.05–0.2 mg·mL^−1^), a significant decrease in biofilm biomass formation was observed. It has also been shown that these concentrations of CAN can negatively affect the expression of genes responsible for QS and biofilm formation. Furthermore, a decrease in bacterial mobility and exopolysaccharide formation was observed [190].

Chadha et al. (2022) examined the synergistic activity of the combination of CNA and gentamicin on quorum sensing in the *P. aeruginosa* (PA01) strain. The QS was significantly reduced, the biofilm structures were eliminated, and their formation was inhibited. All forms of *P. aeruginosa* cell movement have also been suppressed. The anti-virulent effect of this combination was proven in the in vivo model of *C. elegans* tissue infection [191]. The comparable cinnamaldehyde inhibiting properties of biofilm formation, decreased virulence, reduced mobility, and QS of *P. aeruginosa* (PA01) were described in the study by Subhaswaraj et al. (2018) [192].

The antibacterial properties of honey, carvacrol, and cinnamaldehyde, in combination and alone, against *P. aeruginosa* isolates from burn wounds were examined. The control group was treated with imipenem. The average MIC value of three substances was about 430 times lower than the MIC value of IMI. The synergistic activity of these three bioactive compounds has been demonstrated [193]. Ferro et al. (2019) examined the effect of CNA on the healing process of wounds infected with *P. aeruginosa*. It has been demonstrated that the application of CNA at sub-inhibitory concentrations contributed to the inhibition of bacterial biofilm formation and its metabolism, as well as limited the hemolytic activity of the pathogen. It was observed that in the in vivo murine infection model, cinnamaldehyde accelerated wound healing. The decrease in the formation of proinflammatory cytokines (e.g., IL-17), NO, and VEGF (vascular endothelial growth factor) was noted [194].

In the study conducted by Tetard et al. (2019), the exposure of MDR *P. aeruginosa* to high (900 μg·mL^−1^) and sub-inhibitory concentrations of cinnamaldehyde was evaluated. *Pseudomonas* is capable of developing multi-drug resistance through hyperexpression of the efflux pump (e.g., MexAB-OprM, MexXY-OprM) as a result of exposure to sub-inhibitory concentrations of CNA. The study confirmed the increased resistance of *P. aeruginosa* (PA14) to meropenem, ceftazidime, tobramycin, and ciprofloxacin after exposure to CNA [195]. Moreover, the 10 day exposure to a high (900 μg·mL^−1^) concentration of CAN led to the development of mutated isolates that were resistant to numerous drugs (including imipenem and colistin). In addition, the resistance to aztreonam, ticarcillin, colistin, or gentamicin of *P. aeruginosa* clinical isolates (mutants in the area of the DNA repair gene *mutS*) after exposure to CNA increased [196].

##### *Escherichia* *coli*

The He et al. (2019) study evaluated the impact of CNA on cell membrane function and regulation of the expression of key genes in *E. coli* (ATCC 25922 and ATCC 8735). The cinnamaldehyde at various concentrations (multiples of MIC values) showed a direct effect on cell membrane permeability, resulting in cytolysis. In turn, CNA sub-inhibitory concentrations (0.1–0.375 MIC values) inhibited the growth of *E. coli* [197]. Different promising additive or synergistic combinations of CNA and antibiotics used against *E. coli* strains (originally resistant to erythromycin, bacitracin, and others) have been demonstrated. In all tested cases, the addition of CNA led to synergy with erythromycin. However, it did not decrease the MIC value for bacitracin. Moreover, the synergistic activity of tetracycline and CNA against the ATCC 23739 strain as well as the partially synergistic and additive combination of CNA and novobiocin against the ATCC 23739 and 02:0627 strains were observed [198].

The in vivo murine infection model showed that treatment with CNA (60 mg·mL^−1^) improved the survival of laboratory animals with sepsis of *E. coli* (F5) etiology. The postmortem evaluation showed that the internal organs (lungs and kidneys) were less affected by inflammation and damage. The cinnamaldehyde also induced myeloid leukocyte synthesis, showing its significant immunomodulatory effect. The decrease in proinflammatory cytokine secretion resulted in the limitation of the systemic inflammatory response in sepsis, which led to reduced infection mortality [171]. Another study showed the inhibitory effect of CNA (the MIC value = 780 μg·mL^−1^) on the colonisation of the intestinal mucosa in an in vivo mouse model, which is important information regarding the potential for eradication of pathogenic *E. coli* (i.e., EAEC 042) causing diarrhoea. Importantly, the study also showed an effective CNAs safety profile for both Hep-2 cell lines and the in vivo model of the *Tenebrio molitor* larva [199]. Narayanan et al. (2017) assessed the impact of oral supplementation with cinnamaldehyde in the in vivo murine model on the reduction of urinary tract colonisation by *E. coli* UPEC. The postmortem assessment was made after 10 days of the administration of CAN and the infection of animals by the catheter. The evaluation showed a significant decrease in the colonisation of the bladder and urethra by microorganisms. This is a promising alternative treatment for UTIs, as they represent one of the most common infections among people [200]. Another study evaluated the effect of CNA, *p*-coumar acid (CA), and ferulic acid (FA) on the formation of *E. coli* UPEC biofilm on the surface of the urological catheter. The study showed that CNA had the potential to inhibit bacterial biofilm formation over the entire range of its concentration (i.e., from 0.1% to 1.5%), while comparators exhibited this effect only at specific concentrations (0.25% and 0.5% of CA and 0.5% of FA). Only CNA led to the complete inactivation of the biofilm, thus showing the highest activity of all tested compounds [201].

The subsequent research on the possibility of CNA, carvacrol, and eugenol hindering QS activity and reducing the virulence of clinical MDR *E. coli* isolates (including UPEC) was evaluated. The MIC value for CAN (130–260 μg·mL^−1^) was the lowest for all of the tested compounds. Bioactive chemicals applied at sub-inhibitory concentrations contributed significantly to the inhibition of bacterial cell growth. There was also a decrease in bacterial biofilm formation. In addition, the synergistic effect of CNA in combination with tigecycline on UPEC *E. coli* has been demonstrated [202]. Another study showed the high effectiveness of the CNA combination with silver nanoparticles (the MIC value = 0.008–0.016 mg·mL^−1^) against MDR *E. coli* strain EAEC (Enteroaggregative *E. coli*). The high level of systemic safety presented as low cytotoxicity against Hep-2 cells was also detected. The in vivo model of infection on *Galleria melonella* larvae presented a significant decrease in the number of bacteria. The pure cinnamaldehyde obtained similar efficacy to meropenem in an in vivo study [203].

Another study showed that the combination of CAN with allyl isothiocyanate under elevated hydrostatic pressure can increase the effectiveness of both compounds against UPEC *E. coli* and STEC O157:H7 *E. coli* (Enterohaemorrhagic *E. coli*), producing a Shiga toxin [204]. Sheen et al. (2018) have also reported similar results of the combination of CNA with elevated hydrostatic pressure against UPEC and STEC *E. coli* [205]. According to the study by Zhu et al. (2020), the combination of CNA and carvacrol had similar results against O157:H7 *E. coli* [206]. Cinnamaldehyde was the most effective in inhibiting *E. coli* growth and biofilm formation (comparable to eugenol, terpineol, and citronellol). At the cellular level, numerous metabolic dysfunctions of cells have been observed, resulting in a complete lack of ability to form organised structures and significant damage to pathogen cell membranes. Defects in the general cell morphology (i.e., formation of filamentous forms) were observed [207]. Similar findings were obtained in the study by Yossa et al. (2014) on the impact of the combination of CNA and acetic acid on nalidixic acid-resistant *E. coli* (O157: H7) [208].

#### 2.4.2. Biological Activity

As a result of the cinnamaldehyde treatment against *S. aureus*, numerous membrane dysfunctions—wall separation; wall and cell membrane lysis; cytoplasm condensation; and cell morphology disorder—were observed [169]. The ability of CNA to disrupt the metabolism of phosphatidylglycerol and phosphatidylethanolamine is attributed to its antibacterial effect against *S. aureus* and *E. coli*. Eventually, this leads to disruption of the membrane structure and increased permeability. The decrease in the expression of bacterial genes responsible for glycerophospholipid synthesis may be responsible for its activity [209]. The trans-CNA can also act as the repressor of certain genes, among others: *mecA* (which codes for the PBP2a protein that promotes resistance to many β-lactam antibiotics) and *hld* (which is responsible for the formation of MRSA biofilm) [172].

The trans-CNA has shown that it has a negative impact on the expression of coding genes in MRSA, including laminine binding protein (*eno*), elastin binding protein (*ebps*), and fibrinogen binding protein (*fib*). In addition, the trans-CNA also caused inhibition of the expression of the *icaA* and *icaD* genes responsible for the formation of polysaccharide adhesin involved in the formation of biofilm. The ability to form a biofilm was also limited due to the inhibition of gene expression encoding the PIA glucosamine polymer. There was a significant decrease in MRSA metabolic activity under the influence of trans-CNA as well [210]. Moreover, there was a significant decrease in the metabolic activity of bacterial cells (a decrease in ATP formation) and an increase in oxidative stress [173].

Cinnamaldehyde, at the molecular level, can reduce the QS gene expression (Fsr system–*fsrB* and *fsrC* genes) and the *gelE* gene (gelatinase coding gene) in *E. faecalis* strains. Furthermore, a decrease in the expression of the *luxS* gene involved in inter-cellular communication and pathogen virulence regulation was observed [179]. The treatment of *K. pneumoniae* with cinnamaldehyde decreased the expression of *acrB* (the gene encoding the component of the efflux acrAB-TolC pump) and *blaTEM*, *blaSHV*, and *blaCTX-M* (the genes encoding specific β-lactamases) [184]. The inhibitory activity of CNA against *Klebsiella* spp. and *S. aureus* FtsZ protein (regulator for cell divisions) and ATP-synthetase (cellular ATP producer) proves the possible mechanism of its antibacterial activity [211].

The cell division of *A. baumannii* can be blocked by cinnamaldehyde through the inhibition of the FtsZ protein. In in silico docking model, the blocking effect of bromine derivatives on the FtsZ protein with GTP-ase activity was shown [186]. The study by Karumathil et al. (2018) showed that CNA and eugenol molecularly reduced the expression of the following genes in *A. baumannii*: *blaP* (conditioning resistance to β-lactams), *adeA*, *adeB*, and *adeC* (efflux pump genes), and *mdrp* (multi-drug resistance protein) [187]. The decrease in the expression of *A. baumannii* key genes for the formation of the structure of biofilm (i.e., *csuA*, *csuE*, *bfmS*, *bfmR*, *ompa*, *bap*, and *abal*) was also noted after the application of cinnamaldehyde [188]. Another study reported that CNA induced rRNA structure degradation as well as increased the expression of heat shock protein genes (e.g., *groES*, *groEL*, and *danK*) and catalase in *Acinetobacter* spp. cells, which indicates the severity of oxidative stress inside the bacterial cells [212].

The additive effect of CNA with tobramycin has been demonstrated in the field of interference with *P. aeruginosa* (Las, Rhl, QS, and PQS) virulence mechanisms [189]. The cinnamaldehyde provokes in *P. aeruginosa* strains the concentration-dependent degradation of the biofilm structure and reduces the intracellular concentration of bis-(3′-5′)-cyclic dimeric GMP, which controls the formation of biofilm [213]. The CNA also induced the limitation of QS and biofilm formation due to the reduction in the formation of acyl homoserine lactones by *P. aeruginosa*. In addition, there was a significant decrease in the synthesis of key virulence factors of *Pseudomonas* spp., namely: hemolysins, proteases, elastases, alginate, pyocyanin, and rhamnolipid [191]. After the exposure of *P. aeruginosa* cells to cinnamaldehyde, the *exoS* and *ampC* genes in the MDR system have been reduced (6.22 and 2.85 times, respectively) due to the triple combination of substances (CNA, carvacol, and honey) [193].

At the molecular level, the CNA induced a concentration-proportional increase in the formation of peroxide dismutase genes in cells (increased expression of SODa, SODb, and SODc), which suggests the induction of oxidative stress by cinnamaldehyde among *E. coli* cells [214]. The molecular changes induced by CNA in cells of *E. coli* (ATCC 8735) were also described. In the bacterial cell membrane, a decrease in the amount of unsaturated fatty acids and an increase in the content of saturated fatty acids were noted. The cytolysis of bacterial cells was also observed. At the DNA level, CNA caused a change in the structure of nucleic acid that had an effect on the expression of a series of genes [197]. Lin S. et al. (2017) proved in their study that CNA has a significant effect on the expression of almost 126 different *E. coli* genes. The especially important finding is the significantly reduced expression (the restriction of cell mobility) of genes involved in the synthesis of proteins forming bacterial flagella (*flgD*, *flhB*, *fliP*, and *fliR*). Moreover, ATP synthase gene expression disorders have been proven, which may be important in determining the molecular effect of CNA on *E. coli* (energy deficit) [58]. Another study showed (the MIC value = 240 μg·L^−1^) the development of oxidative stress conditions, carbohydrate metabolism disorders (pentozo-phosphate pathway), as well as translation defects. Additionally, the conversion of the CNA to the acid aldehyde group generated acid stress at the cellular level, which was considered the main pathogenic agent for microorganisms [215]. The molecular analysis identified a decrease in the expression of the *luxS* gene, which is important in quorum sensing. The reduced expression of *fimA* (adhesion fimbria) and *fliC* (flagella) genes was observed [202].

### 2.5. Ellagic Acid (EA)

**Figure 9:**
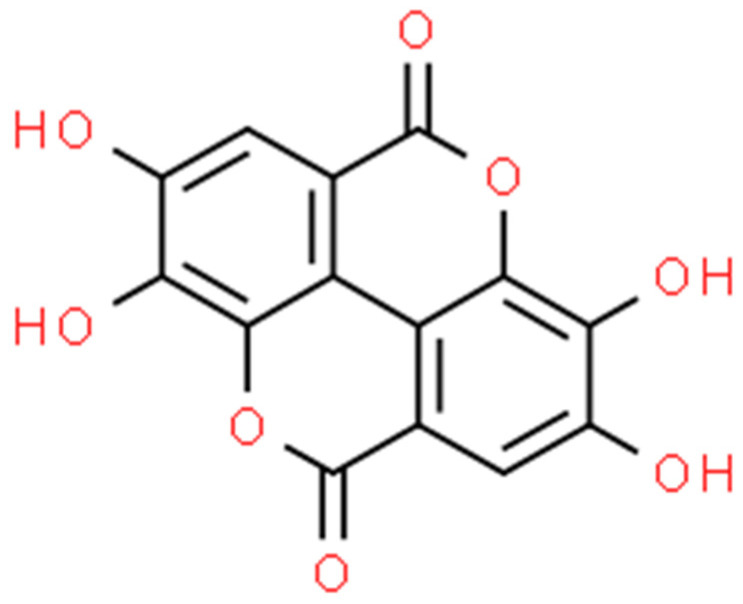
Ellagic acid structure (ChemSpider database).

**Figure 10:**
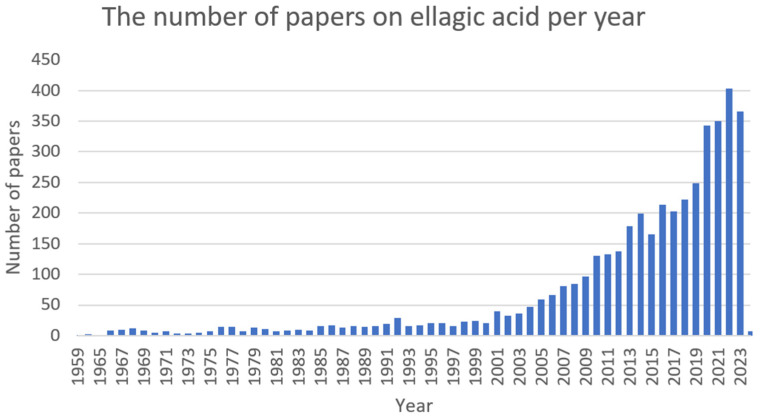
The number of papers on ellagic acid per year (PubMed database).

Ellagic acid (Figure 9) is a bioactive polyphenolic compound that can be extracted from numerous natural sources, such as oak bark, valonea, pomegranate (*Punica granatum* L.), divi-divi (*Caesalpinia coriaria*), myrobalan (*Terminalia catappa*), and algarrobilla (*Prosopis humilis*) [216,217,218]. Ellagic acid in plants is predominately found ester-linked to sugars in the composition of hydrolysable tannins called ellagitannins [219]. In recent years, it has been attracting great attention (Figure 10) due to its distinct antioxidant [220], anti-inflammatory [221], antifibrotic [222], antimutagenic [223], antiproliferative [224], neuroprotective [225], hepatoprotective [226], cardioprotective [227], wound-healing [228], osteogenic [229], antimicrobial [230], antiviral [231], and antiparasitic [232] activities.

#### 2.5.1. Antimicrobial Activity

##### *Staphylococcus* *aureus*

The study by Tavares et al. (2018) determined the antibacterial activity of chitosan, zein, and ellagic acid (separately and in combinations) against various bacterial pathogens. The EA showed the MIC and MBC values at 0.104 and 0.291 mg·mL^−1^ against *S. aureus*, respectively. High activity against *P. aeruginosa* has been proven (the MIC value = 0.208 mg·mL^−1^ and the MBC value = 0.416 mg·mL^−1^). After the combination of EA with chitosan, a significant decrease in their MIC was observed, which demonstrates their synergistic effect [233]. The reduction of the number of bacteria colonising the wound (including *E. coli*, *S. aureus*, and *P. aeruginosa*) was observed after the treatment with EA nanoparticles in a scaffolding film of chitosan, zein, and gelatine. The in vivo model demonstrated that this type of dressing accelerates and enhances wound healing [234]. This study confirms previous research [235,236] conducted by this author. Another study showed that hydrogels containing EA have antibacterial activity against *S. aureus*, *E. coli*, and *P. aeruginosa*. The growth inhibition zone for *S. aureus* was 29 mm, while the positive control, consisting of cefepime (1 mg·mL^−1^), reached 32 mm, which proves the great properties of this natural compound [237].

In the newly published report, the assessment of EA (the MIC value = 1024 μg·mL^−1^) and gallic acid in overcoming the resistance of *S. aureus* strains (IS-58 and K2068) forming efflux pumps, i.e., TetK (tetracycline resistance) and MepA, was conducted. The in vivo model of *Drosophila melanogaster* determined the low toxicity of both compounds. The combination of both substances with tetracycline resulted in a reduction of half of the MIC value (from 256 to 128 μg·mL^−1^) against *S. aureus* (IS-58). In turn, the combination of compounds with ciprofloxacin also led to a decrease in its MIC against *S. aureus* (K2068). Preliminary research suggested that EA is not likely to have an inhibitory effect on efflux pumps in contrast to gallic acid (a TetK inhibitor) [238]. According to the study conducted by Vigbedor et al. (2022), the *Afzelia africana* root extract contains 3,5-di-O-methyl ellagic acid that exhibits a wide range of antibacterial properties. The compound in the concentration range of 6.25–1.56 mg·mL^−1^ inhibited bacterial growth by 40–100%. The bactericidal effect is dependent on concentration, with the greatest effectiveness against *E. coli*. The inhibition of MRSA growth between 49.1% and 27.8% at the tested concentration range (6.25–0.2 mg·mL^−1^) of EA was noted [239].

The study by Dahash et al. (2021) presented a report on the potential synergy of moxifloxacin (MOC) with EA (extracted from pomegranate) against MRSA. The study showed that the MIC value of the combination amounted to 1.22 ± 0.85 mg·mL^−1^, while the MIC value of MOC alone was approximately 2.70 mg·mL^−1^. There was also a significant reduction in the MIC value of the extract containing ellagic acid. The fact that MRSA is a common hospital pathogen responsible for the infection and still has significant mortality makes all of these findings vitally crucial [240]. The antibacterial activity of EA against numerous Gram-negative and Gram-positive pathogens, including *P. aeruginosa* (the MIC value = 500 μg·mL^−1^), *Enterobacter agglomerans*, *Cutibacterium acnes*, and *S. aureus* (the MIC value = 15.6 μg·mL^−1^), along with MDR strains, was confirmed [241]. Fontaine et al. (2017) examined the effect of xyloside and rhamnoside of EA on the ability of MSSA *S. aureus* (isolated from bone and bone marrow infection and mutant in terms of the *sarA* gene-impaired biofilm formation ability) to form biofilm. The EA xyloside and ramnoside inhibited biofilm formation. However, only ramnoside (MBIC_50_ = 64 μg·mL^−1^, MBIC_90_ = 128 μg·mL^−1^, the minimal biofilm inhibition concentration 50% and 90%, respectively) decreased in 90% the ability to create a biofilm by *S. aureus*. The study proved that the rhamnoside EA can be considered an inhibitor of *S. aureus* biofilm formation [242].

##### *Enterococcus* spp.

The Ekrikaya et al. (2021) study evaluated the impact of EA (present in blackberry and raspberry extract) silver nanoparticles [Ag^+^] against *E. faecalis* (ATCC 29212) and other organisms. Nanoparticles based on the raspberry extract showed significant antibacterial properties and the eradication of nearly 85% of the *E. faecalis* population at a concentration of 100 ppm. These results are similar to those of products used in dental disinfectants, i.e., 80% of eradication for chlorhexidine solution (1.5%) and 95% of eradication for NaOCl solution (5%). It is worth noting that extract (10%) without NPs increased eradication by 30% of *Enterococcal* cells, which definitely shows a beneficial interaction with Ag^+^ nanoparticles [243].

##### *Klebsiella* *pneumoniae*

Few findings on the activity of EA against *K. pneumoniae* come from the article Vigbedor et al. (2022) that 3,3′-di-O-methyl ellagic acid was active against *K. pneumoniae* (NCTC13440). The ellagic acid in the concentration range from 6.25 to 0.20 mg·mL^−1^ showed inhibition of pathogen growth (the MIC value = 2.5 mg·mL^−1^, the MBC value = 5 mg·mL^−1^) from 49.1% up to 27.8% [239].

##### *Acinetobacter* *baumannii*

Based on the immunocompromised murine in vivo model, the ability of EA in the liposomal form against MDR *A. baumannii* (ATCC 19606) to inhibit the growth of the pathogens The MIC value was determined at 64 μg·mL^−1^ (inactivation of 99.9% bacterial inoculum within 24 h). The compound showed a concentration-dependent ability to reduce bacterial abundance. In comparison to the control group, the EA at a concentration of 32 μg·mL^−1^ limited the biofilm formation to 72.3%, which proved the good anti-biofilm properties of the ellagic acid. The in vivo model showed that *A. baumannii* infection in mice with pharmacologically induced immunosuppression can be successfully cured using liposomal ellagic acid. Furthermore, this study also demonstrated the activity of EA against the formation of *A*. *baumannii* biofilm. Taking into account the low solubility of EA in water, the authors prepared lipid-based nanoparticle (liposomal) formulations of EA (EA-liposomes) and assessed their effectiveness in the treatment of bacterial infection. The observed increased survival rate (60%) and decreased bacterial load in the lungs, as well as restored liver (AST and ALT) and kidney (BUN and creatine) function parameters, were observed in mice after the use of EA-liposomes (100 mg·kg^−1^). The bronchoalveolar fluid (BALF) from infected mice contained greater quantities of IL-6, IL-1b, and TNF-a, which were significantly alleviated in mice treated with EA. These results support the possible implication of EA-liposomes in the treatment of *A*. *baumannii* infection, especially in immunocompromised mice [244].

##### *Pseudomonas* *aeruginosa*

The anti-*Pseudomonas* activity of ellagic acid (the MIC value = 0.208 mg·mL^−1^ and the MBC value = 0.416 mg·mL^−1^) has been proven in a series of studies conducted by Tavares et al. [233,234,235,236]. The confirmed properties of EA to combat *P. aeruginosa* were provided by Yu et al. (2023). The hydrogel products containing EA showed antibacterial properties against *Pseudomonas* (the growth inhibition zone was 14 mm, while the positive control (cefepime) was 34 mm) [237]. In the Bai et al. (2022) study, the EA at 50 μmol prolonged the survival of a model nematode (*C. elegans*) with *P. aeruginosa* infection by 79.07% and reduced the number of pathogens in the model organism tissues [245]. Another study also showed the positive effect of 3,3′-di-O-methyl ellagic acid on *P. aeruginosa*. This compound (the MIC value = 2.5 mg·mL^−1^, the MBC value = 5 mg·mL^−1^), at a concentration range of 6.25–0.20 mg·mL^−1^, caused growth inhibition by 53.9–34.9%, respectively [239].

In the study by Al-Mugdadi et al. (2019), ellagic acid showed antibacterial activity against *P. aeruginosa* (the MIC value = 500 μg·mL^−1^) [241]. Another study identified the effects of EA, epigallocatechin, and tannic acid on *P. aeruginosa* (PAO1) in the inhibition of biofilm formation and the reduction of bacterial adhesive capacity. Strong activity in the field of inhibition of bacterial growth (depending on the concentration of EA) has been proven. In the concentration range of 20–800 mg·L^−1^ of ellagic acid, inhibition of the biofilm formation of *P. aeruginosa* was observed. For instance, the biofilm creation oscillated at around 58.47% at a concentration of 20 mg·L^−1^ [246]. The study on the inhibition of QS of *P. aeruginosa* (PAO1) was examined by Sarabhai et al. (2015). The authors showed that a concentration of 5 mg·mL^−1^ of the ellagic acid derivative can inhibit the synthesis of alginate (a polysaccharide involved in the formation of biofilm) by more than 50%. At the same concentration, the biofilm formation was blocked by 85%. Furthermore, the combination of tobramycin (20 μg·mL^−1^) with derivatives of EA derivatives (1 mg·mL^−1^) increased the inhibitory effect of the antibiotic on the biofilm (from 60% to more than 80%) [247]. In addition, exposure to EA has been shown to potentiate the bactericidal effect of piperacillin against *P. aeruginosa* (PAO1) [248].

##### *Escherichia* *coli*

The study by Yu et al. (2023) showed the effect of EA (in the form of hydrogel) on *E. coli* in terms of the limitation of growth capacity (the growth inhibition zone was 31 mm, while for the control group (cefepime), it was 35 mm) [237]. The reports on the 3,3′-di-O-methyl ellagic acid activity (the MIC50 = 0.5 mg·mL^−1^, the MBC = 2.5 mg·mL^−1^, the MIC value > 2.5 mg·mL^−1^) against *E. coli* were investigated by Vigbedor et al. (2022). The inhibition of bacterial growth at 92.5–35.4% was achieved with the ellagic acid concentrations ranging from 6.25 to 0.20 mg·mL^−1^, respectively [239]. The effectiveness of EA-coated gallium nanoparticles against *E. coli* and their comparison with gentamicin (GEN) were also evaluated. The tests were conducted with the use of the disc diffusion method, and the growth inhibition zone for 1 mg·mL^−1^ of EA nanoparticles was determined at 12 mm, while for GEN at a concentration of 4 μg·mL^−1^, the growth inhibition zone was 30 mm [249]. The study by Jenic et al. (2021) evaluated the effects of EA and other naturally occurring substances on MDR *E. coli* (NCTC 13400) as well as the interactions between substances and antibiotics classically used for the treatment of *E. coli* infections. Treatment with the combination of EA and tetracycline resulted in a decrease in the MIC value of tetracycline from 32 to 16 μg·mL^−1^ after 8 h and from 64 to 32 μg·mL^−1^ after 21 h. The ellagic acid in the combination with chloramphenicol led to a 4-fold decrease in the MIC value for the antibiotic against *E. coli* (from 8 μg·mL^−1^ to 2 μg·mL^−1^) [250].

#### 2.5.2. Biological Activity

The use of ellagic acid against *P. aeruginosa* can reduce the formation of EPS (exopolysaccharides) related to the formation of biofilm. There was no evidence for the inhibition of biofilm dispersion [246]. The derivatives of EA were evaluated for their ability to inhibit polyphosphate kinase 1 (PPK1), an enzyme that regulates the virulence of *P. aeruginosa* PAO1 (isolates from burn wounds and septic infections) and limits oxidative stress. The concentration of 0.5 mg·mL^−1^ can cause the complete inhibition of PPK1, which resulted in the impaired expression of *rpoS* (the main stress response regulator) by over 94%. There was a significant decrease in catalase activity and peroxide dismutase, which led to cell death as a result of ROS-induced oxidative stress [248]. The decrease in the expression of key virulence genes of *P. aeruginosa* PAO1 (such as *lasIR* and *rhlIR*) under the influence of EA was detected in the study by Sarabhai et al. (2013). The synthesis of crucial virulence factors, such as pyocyanin, elastase, rhamnolipids, alginate, and proteases, has also been reduced [247].

According to the Ratti et al. (2023) study, the molecular impact of EA on the WrbA protein expression in *E. coli* can cause a decrease in pathogen biofilm formation as well as promote oxidative stress. The in silico model showed the ability of EA to directly block the WrbA protein, which leads to increased oxidative stress by ROS. Additionally, a highly effective EA anti-biofilm property profile was demonstrated. This active compound, at concentrations of 0.5–500 μmol, stimulated inhibition of biofilm formation at a level from 60 to 94% [251].

### 2.6. Proanthocyanidin (PAC)

**Figure 11:**
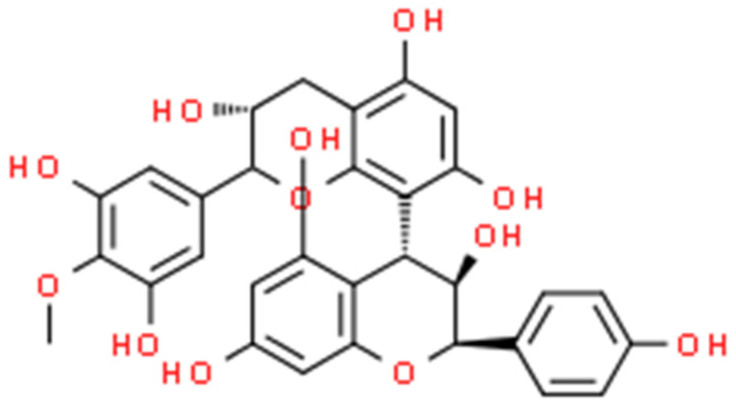
Proanthocyanidin structure (ChemSpider database).

**Figure 12:**
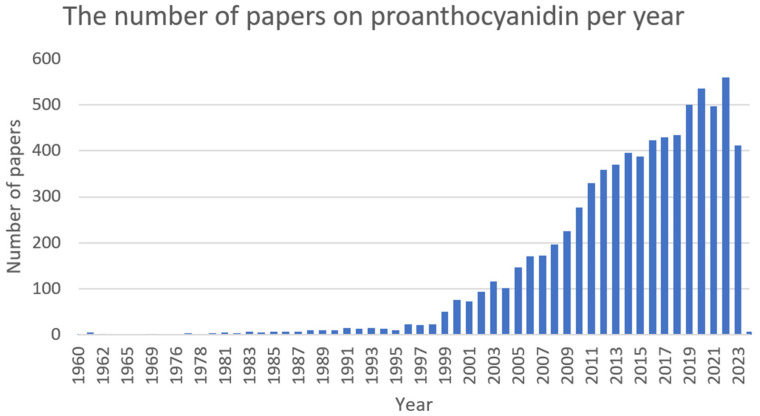
The number of papers on proanthocyanidin per year (PubMed database).

Proanthocyanidins (Figure 11) are condensed tannins obtained by the polymerisation of flavan-3-ol as a structural unit via a C–C bond. On the basis of the degree of polymerisation, they can be divided into oligomeric and polymeric forms. Proanthocyanidins are the most abundant in fruits but can also be found in vegetables and coloured grains. Among the sources with a higher concentration of these compounds are grape seeds (*Vitis* L.), cranberries (*Vaccinium* L.), Chinese wolfberries (*Lycium chinense*), red goji berries (*Lycium barbarum*), pomegranates (*Punica granatum*), and red rice (*Oryza* L.) [252,253,254]. Presumably, they represent the second most abundant class of polyphenols in the plant kingdom after lignin [255]. Numerous in vitro and in vivo studies have confirmed the beneficial human and animal health properties of proanthocyanidins. They possess anti-inflammatory [256], antioxidant [257], antidiabetic [258], neuroprotective [259], cardioprotective [260], antimicrobial [261], immune regulatory [262,263], anti-obesity [264], hypoglycemic [265], hypolipidemic [266], and anticancer [267] activities (Figure 12).

#### 2.6.1. Antimicrobial Activity

##### *Staphylococcus* spp.

The effect of proanthocyanidin, which can be obtained from the *Camelia sinensis* plant, on MRSA and MSSA *S. aureus* was examined. This study was carried out in four proanthocyanidin F1–F4 fractions. Fraction 1 contained monomeric flavan-3-ols; fraction 2 consisted of monomeric flavan-3-ols and PAs; and fractions 3 and 4 contained only PACs. Fraction 4 showed the highest activity against standard *S. aureus* strains (ATCC25923 and NCTC 6571). The MIC values of the tested strains (including MRSA and MSSA) were between 512 and 1024 mg·mL^−1^, suggesting moderate antimicrobial activity of the tested compound [268].

The activity of proanthocyanidin irradiated with a 405 nm laser beam against *S. aureus* was also evaluated. The CFU reductions of >5 log were observed within half an hour. The PAC generates ROS during the treatment, which allows the killing of the microorganisms. The separate action of the laser and proanthocyanidin did not give a satisfactory effect, but in combination, it gave a sufficient result. These properties may suggest the possibility of using this treatment in the disinfection process [269].

Proanthocyanidin, isolated from *Blechnum orientalis*, showed bactericidal activity against Gram-positive bacteria (*Bacillus cereus* ATCC14579, *Micrococcus luteus* ATCC4698, MSSA ATCC25923, MRSA ATCC33591, and *S. epidermidis*). The antibacterial effect was measured by disc diffusion and broth dilution tests. On this basis, the MIC value was determined to be 31.3–62.5 µg·mL^−1^, and the MBC value was 31.3–62.5 µg·mL^−1^. This study also paid particular attention to the demonstrated anticancer activity of HT29 cells [270].

Interestingly, attempts have been made to combine PAC with silver nitrate (1%) and ferric chloride solution (1%) for potential antimicrobial and anticancer properties. The test was carried out using the diffusion technique in an agar well. After 24 h of incubation at 37 °C, the zones of inhibition of *S. aureus*, *E. coli*, and *P. aeruginosa* were measured by comparing the tested compound with ciprofloxacin. The zone of inhibition was significantly smaller compared to the antibiotic, suggesting the lack of clinical application of this combination in the fight against microorganisms [271].

##### *Enterococcus* spp.

The effect of PAC against *E. faecalis* on dentin blocks infected with a 1-week biofilm was assessed. For comparison, the following: sterile water, chlorhexidine (2%), PAC (2%), PAC (5%), and PAC (10%) were used. A significant increase in the number of dead *E. faecalis* cells was demonstrated in tests with PAC and chlorhexidine. Chlorhexidine showed a bactericidal effect but had no effect on the demineralised collagen contained in dentin, unlike PAC, which showed a concentration-dependent effect. PAC caused an increase in collagen biostability and elasticity. Based on these studies, it can be assumed that PAC is a possible alternative to other antibacterial agents in endodontics [272].

##### *Escherichia* *coli*

A combination of proanthocyanidin and chitosan has been created. The influence of these compounds on the colonisation of the intestine by *E. coli* was examined. Various quantitative combinations of these substances were used to assess the agglutination of the extraintestinal *E. coli* strain EPEC. A significant reduction in the invasiveness of these microorganisms has been demonstrated, which is influenced by the ability of combining these compounds to agglutinate virulence factors. Synergism improves the effect of proanthocyanidin, which also acts on the bacterial cell surface itself [273]. A similar result was obtained in a study in which chitosan particles were combined with proanthocyanidin biopolymers created by ionotropic gelation. Interestingly, proanthocyanidin alone reduced the ability of the EPEC strain to invade the intestinal epithelium by 82% (for a concentration of 66 μg of gallic acid equivalent), while the combination of compounds reduced it by 80%, suggesting that PA is responsible for the antimicrobial effects [274].

The different interflavan bond ratios can occur in various plants. Type A predominates in cranberries, while type B predominates in apples. This translates into antibacterial activity against EPEC *E. coli* strains, as proven by the research of Feliciano et al. (2014). Plants with type A bonds have a greater impact on inhibiting the invasion of the intestinal epithelium by strains and also cause agglutination [275].

##### *Pseudomonas* *aeruginosa*

The synergism of ciprofloxacin with the active fraction of Proanthocyanidin *Vaccinum macrocapron* (PAF) against *P. aeruginosa* was tested. The MIC value for PAF was determined to be 25 mg·mL^−1^ for the PAO1 strain. The pathogenesis of *P. aeruginosa* is related to the phenomenon of quorum sensing, which involves bacteria communicating with each other using chemical compounds. It is important to search for inhibitors of this phenomenon. This combination significantly reduced biofilm growth and virulence factors. Among the tested virulence factors, which included elastase, protease, cell-associated hemolysin, cell-free hemolysin, pyochelin, rhamnolipid, and alginate, only pyochelin and protease were reduced. This combination offers a good perspective in the treatment of infections caused by *P. aeruginosa* [276].

A combination of proanthocyanidin-chitosan and gentamicin has also been developed. The effect of this compound on *S. aureus*, *E. coli*, and *P. aeruginosa* was tested. The bactericidal activity of this combination was found to be higher than that of gentamicin alone. For *E. coli*, the CFU·mL^−1^ was reduced by 10%, for *P. aeruginosa* by 64%, and for *S. aureus* by 36% compared to the negative control. The ability of the compound to agglutinate the above-mentioned microorganisms was also confirmed, with particular emphasis on the dose-dependent increase in *E. coli* agglutination. This relationship was also observed in *P. aeruginosa*, while agglutination of *S. aureus* did not show such a relationship. The mobility of these bacteria also decreased. However, the test results showed that the MIC and MBC values were greater or equal for the tested combination than for gentamicin alone [261].

The activity of proanthocyanidins isolated from the above-mentioned cranberry, *Vaccinum macrocarpon* alone as well as in combination with various antibiotics, was tested. Increased sulfamethoxazole activity of gentamycin, kanamycin, nitrofurantoin, tetracycline, and azithromycin was observed. This phenomenon allows for a significant reduction in the amount of antibiotic administered against the tested bacterial strains, i.e., *E. coli* (CFT073), *P. mirabilis* (HI4320), and *P. aeruginosa* (PA14). The effect of trimethoprim and fosfomycin against the *P. mirabilis* strain was also improved [277].

#### 2.6.2. Biological Activity

Proanthocyanidin has the ability to create reactive oxygen species after irradiation with a 405 nm laser beam [269]. It was also condensed with cinnamaldehyde and its four derivatives. It has been proven that the combination has a greater affinity for the fimbriae of EPEC strains, which suggests better effectiveness in the agglutination process of these microorganisms at the designated equivalent concentration of 200 µg·mL^−1^. Cinnamaldehydes themselves do not have the ability to agglutinate microorganisms [278]. A-type bonds characteristic of proanthocyanidin affect the *fliC* gene, which is responsible for the production of flagellin, which significantly limits the mobility of the microorganism (*E. coli* CFT073) [275]. The combination of ciprofloxacin with the active fraction of proanthocyanidin, *Vaccinum macrocarcolion*, was used in studies on the antimicrobial activity of *P. aeruginosa*. This resulted in inhibition of the production of acyl-hoserine lactone and *Pseudomonas* quinolone signal, which are among the molecules responsible for quorum sensing [276].

### 2.7. Sanguinarine (SG)

**Figure 13:**
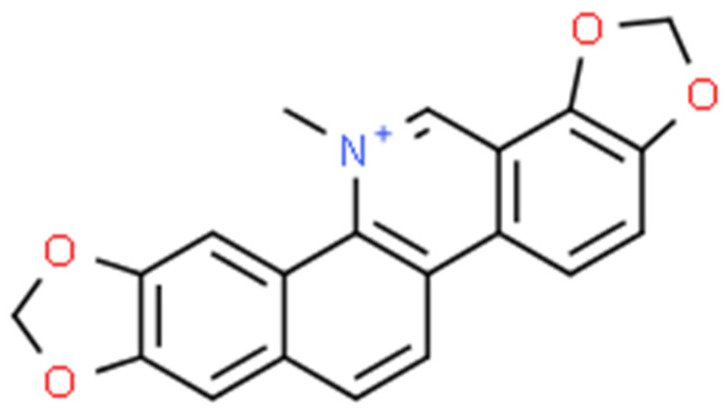
Sanguinarine structure (ChemSpider database).

Sanguinarine (Figure 13), found in plants such as *Sanguinaria canadensis* (blood root), *Poppy fumaria*, *Bocconia frutescens*, *Chelidonium majus*, and *Macleya cordata* [279], is the most commonly used benzophenanthridine alkaloid. It is a benzophenanthridine structural homolog of chelerythrine [280].

**Figure 14:**
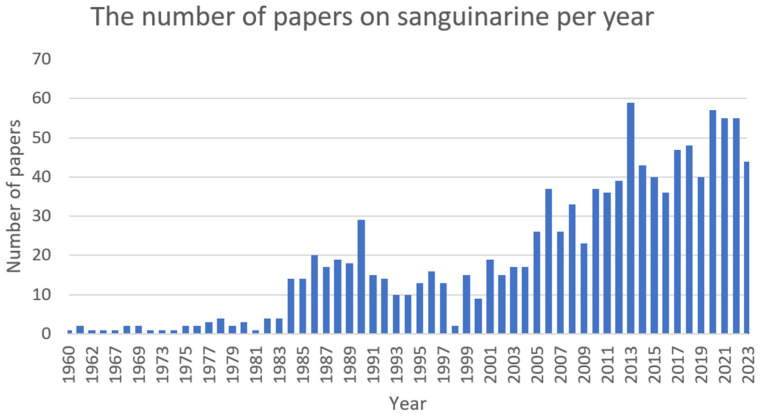
The number of papers on sanguinarine per year (PubMed database).

It exhibits antimicrobial [281], antioxidant [282], anti-inflammatory [283], cytotoxic [284], cytostatic [285], cardiac [286], and antiplatelet [287] activity (Figure 14).

#### 2.7.1. Antimicrobial Activity

##### *Staphylococcus* spp.

Sanguinarine exhibits activity against MRSA and *E. coli* microorganisms and has anti-inflammatory, anti-cancer, and antimicrobial properties. In the study of hydrogel dressings containing sanguinarine, the effectiveness of the compound in the release of autolytic enzymes of bacteria associated with the cell wall, leading to its lysis, was measured at an MIC of 3.12 μg·mL^−1^ to 6.25 μg·mL^−1^ [288].

The effect of sanguinarine chloride hydrate (SGCH) (obtained from the *Macley cordata* plant), which is a form of sanguinarine hydrochloride, was investigated. The activity of the tested compound correlated positively with the increase in SGCH concentration. For SGCH of 1000 μg·mL^−1^, the inhibition zone was 15.67 mm, and the compound was assessed as medium-sensitive. The MIC value against *S. aureus* amounted to 128 µg·mL^−1^, and the MBC was 256 µg·mL^−1^. At 8 MIC, after 24 h of culture, the colonies disappeared, indicating the killing properties of SGC at a given concentration. Sanguinarine hydrochloride affects the morphology of cells, the shapes of which become irregular and their surfaces rough [289]. 

The synergism of sanguinarine with the antibiotics streptomycin and EDTA, showing a better effect on Gram-positive (the MIC value = 0.5–8 μg·mL^−1^) than Gram-negative (the MIC value = 4–128 μg·mL^−1^) bacteria, was observed. *A. baumannii*, MRSA, VRE, and *E. coli* that were resistant to streptomycin showed sensitivity to sanguinarine. Due to the combination of different substances, the effective doses of the drugs were lowered. Sanguinarine with a value of 16 MIC reduced the number of MRSA, *E. coli*, and *K. pneumoniae* bacteria by up to 1000 times [290].

##### *Enterococcus* spp.

The effect of sanguinarine in combination with ciprofloxacin, nitroxoline, and zinc pyrithione in the treatment of bacterial diseases with diarrhoea and the influence of this combination on the intestinal flora were described. The diarrhoea-causing organisms used in this study included *B. cereus*, *E. faecalis*, *L. monocytogenes*, *S. flexneri*, and *V. parahaemolyticus*. The findings showed a bacteriostatic effect against the mentioned microorganisms but did not prove a bactericidal effect. Interestingly, an antagonistic effect against bifidobacteria (*B. adolescentis*, *B. animalis*, and *B. breve*) was found, which suggests a beneficial effect on the intestines during treatment [291].

##### *Escherichia* *coli*

The activity of sanguinarine in the treatment of ulcerative colitis in mice was investigated. It showed anti-inflammatory effects by lowering the concentration of pro-inflammatory cytokines and, at the same time, lowering the concentration of anti-inflammatory cytokines. The increased expression of NLRP3 (a complex associated with inflammation) was detected in mice treated with sodium dextran. Sanguinarine is an inhibitor of the NLRP3 inflammasome as well as IL-1beta and caspase-1, which significantly improves the condition of the large intestine. The number of *Escherichia* spp., *Helicobacter* spp., and *Shigella* spp. has been reduced [292]. Due to the antimicrobial effect of sanguinarine, hydrogel dressings containing the tested compound at a concentration of 0.2 µg·mL^−1^ and BmKn2 at a concentration of 10 µg·mL^−1^ were tested. The synergistic effect contributes to the elimination of nearly 100% of *E. coli* and 100% of *S. aureus*, which suggests the possibility of including a hydrogel dressing with sanguinarine in the treatment of infections within the surgical site [293].

##### *Acinetobacter* *baumannii*

Sanguinarine, in combination with aminoglycosides, has sufficient antimicrobial activity due to the synergistic action of this antibiotic. This drug increases the permeability of the membrane, which improves the penetration of sanguinarine. After entering the cell, it generates ROS production, which may contribute to cell death. No similar effect was observed when SG was combined with beta-lactams or fluoroquinolones. This connection works for, among others, *E. coli*, *A. baumannii*, *K. pneumoniae*, and *P. aeruginosa* [294].

#### 2.7.2. Biological Activity

High concentrations of sanguinarine hydrochloride stimulate the production of ROS by *S. aureus*, which causes oxidative damage to the cell. Membrane permeability increases, as indicated by the leakage of K^+^, Na^+^, Ca^2+^, and Mg^2+^ ions [289]. Moreover, in combination with an aminoglycoside, reactive oxygen species are also produced. The antibiotic improves the penetration of sanguinarine, which causes oxidative stress inside the cell [294]. In the study conducted by Falchi et al. (2021), the effect of sanguinarine on *P. aeruginosa* (PA01) was investigated. Sanguinarine causes the blocking of the KguD or KguK enzymes, which leads to the reduction of bacterial growth. This is due to the inability to use 2-ketogluconate for the synthesis of glucose-6-phosphate, which is responsible for the previously mentioned enzymes located in the cytoplasm [295].

## 3. Materials and Methods

This review refers to research papers from the Scopus, Web of Science, PubMed, ScienceDirect, and Google Scholar databases published between 1999 and 2023. In total, 286 papers were cited. In the database search, the following keywords were checked: ’berberine’, ‘catechin’, ‘chelerythrine’, ‘cinnamaldehyde’, ‘ellagic acid’, ‘proanthocyanidin’, ‘sanguinarine’, ‘*Staphylococcus*’, ‘*Enterococcus*’, ‘*Klebsiella*’, ‘*Escherichia*’, ‘*Pseudomonas*’, ‘*Acinetobacter*’ in the topic and abstract of articles. Table 1 consists of a list of plant compounds discussed in the current study and their activities. Table 2 lists, alphabetically, the compounds considered and their antibiofilm activity.

## 4. Conclusions

The rising prevalence of drug-resistant bacteria poses a considerable risk to human and animal health. In view of the decreasing effectiveness of the antibiotics currently used in medicine, it is of utmost importance to search for novel and nature-based solutions. One of the approaches may be the use of plant-derived bioactive compounds that have been known to exhibit significant health benefits, as confirmed by various studies using human and animal models. They exert low toxicity towards healthy cells and have well-documented, among others, anticancer, anti-inflammatory, antibacterial, antiviral, antioxidant, cholesterol-lowering, antidiabetic, antiobesity, hypolipidemic, hepatoprotective, antihypertensive, antidepressant, antidiarrheal, and anti-neurological activities. The current situation in the post-COVID era, which is associated with increased resistance of the strains responsible for infections, actually demands the need to expand research in this direction. Particularly desirable are studies related to the absorption and bioavailability in the human organism of these promising compounds. There are a growing number of articles related to this topic, but the major limitation of these studies is that they were conducted in vitro or in vivo on animal models, so the mechanisms of action may be different for the healing of infected human wounds. Notwithstanding that this field still requires intensive research, it does not alter the fact that plants will become a major point of interest for the pharmaceutical industry due to their capacity to synthesise organic compounds with specific pharmacological activity and will represent potential alternatives to antibiotics for fighting infectious conditions. The development in this area is also driven by the growing demand of consumers for natural remedies; thus, the need for botanical-based antimicrobials will continue to grow. For this reason, research focused on the identification, application, and verification of new and existing natural antimicrobials to protect health is critical. This knowledge can be valuable for the progress of future therapeutic strategies.

## Figures and Tables

**Table 1:** The list of substances and their molecular antibacterial activity (alphabetically).

Substance	Antibacterial Effect	References
**Berberine**	Inhibition of D-Ala-D-Ala synthesis Inhibition of metabolism of nucleic acids and aminosaccharides Generation of oxidative stress with inhibition of antioxidant synthesis	[99]
	FtsZ protein inhibition	[75]
	acrAB-TolC efflux pump overexpression	[82]
	Nucleic acid metabolism dysfunction Cellular wall lysis Inhibition of transmembrane transport	[101]
	Inhibition of bacterial DNA synthesis	[89]
	Inhibition of the MexXY-OprM efflux pump	[91,94,95,96,103]
**Catechin**	Increase in oxidative stress due to the generation of ROS in bacterial cells Degradation of DNA polymers	[140]
	Inhibition of *norA*, *norC*, and *abcA* MRSA efflux pump gene expression	[123]
	Direct inhibition of MurA, GBAP, and gelatinase	[127]
**Chelerythrine**	Increase in membrane permeability	[148]
**Cinnamaldehyde**	Inhibition of phosphatidylglycerol and phosphatidylethanolamine metabolism Increase in cell membrane permeability	[209]
	Inhibition of *mecA* and *hld* gene expression	[172]
	Inhibition of (*eno*), (*ebps*), and (*fib*) gene expression	[210]
	Inhibition of *acrB* gene expression Inhibition of _bla_TEM, _bla_SHV, and _bla_CTX-M genes expression	[184]
	Inhibition of the FtsZ protein	[186,211]
	Inhibition of: _bla_P, *adeA*, *adeB*, *adeC*, *mdrp* gene expression	[187]
	Inhibition of Las and Rhl virulence factors	[189]
	Inhibition of *exoS* and *ampC* gene expression	[193]
	Inhibition of *flgD*, *flhB*, *fliP*, and *fliR* gene expression	[58]
**Ellagic acid**	Direct inhibition of polyphosphate kinase 1 Inhibition of *rpoS* gene expression Decrease in catalase and peroxide dismutase activity Cell damage due to ROS accumulation	[248]
	Cell damage due to ROS accumulation	[251]
**Proanthocyanidin**	Bacterial agglutination due to affinity for the fimbriae	[278]
**Sanguinarine**	Cell damage due to ROS generation Increase of membrane permeability	[289]
	Direct inhibition of KguD or KguK enzymes Inhibition of cellular glucose–6-phosphate synthesis	[295]

**Table 2:** The list of substances and their molecular antibiofilm activity (alphabetically).

Substance	Antibiofilm Effect	References
**Berberine**	Inhibition of *agrA-D* gene expression	[68]
	Inhibition of PSM aggregation	[74]
	Inhibition of expression of sortase genes (*srtA* and *esp*)	[80]
	Decrease in the production of alginate	[93]
	Inhibition of *sarA*, *cidA*, and *icaA* gene expression	[100]
	Inhibition of *luxS*, *pfS*, *hflX*, *ftsQ*, *uxS*, and *ftsE* gene expression	[102]
	Inhibition of the exopolysaccharide synthesis	[90]
	Inhibition of QS signalling by docking at LasR and RhlR	[92,105]
**Catechin**	Inhibition of the GBAP protein	[127]
	Direct inhibition of the BfmR protein	[135]
	Inhibition of *lasR* and *lasI* gene expression	[137]
	Direct inhibition of the LasR factor	[137]
**Cinnamaldehyde**	Increased expression of the AI-2 factor	[168]
	Reduction of bacterial exopolysaccharide formation	[179]
	Direct inhibition of ESP protein	[180]
	Decreased level of QS involved genes encoding: hemolysins, proteases, elastases, alginate, pyrocyanin, and ramnolipid expression	[190,191,192]
	Inhibition of exopolysaccharide synthesis	[190]
	Inhibition of *icaA* and *icaD gene* expression	[173]
	Inhibition of gene-encoding PIA glucosamine polymer expression	[173]
	Inhibition of *fsrB*, *fsrC*, *luxS*, and *gelE* gene expression	[179]
	Inhibition of: *csuA*, *csuE*, *bfmS*, *bfmR*, *ompa*, *bap*, *abal* genes expression	[188]
	Reduction of the intracellular concentration of bis-(3′-5′)-cyclic dimeric GMP	[213]
	Reduction in the formation of acyl homoserine lactones	[191]
	Inhibition of *luxS*, *fimA*, and *fliC* gene expression	[202]
**Ellagic acid**	Inhibition of *lasIR* and *rhlIR* gene expression	[247]
	Inhibition of the formation of exopolysaccharides	[246]
	Direct inhibition of WrbA protein	[251]
	Inhibition of the synthesis of alginate	[248]
**Proanthocyanidine**	Reduction of expression of genes that encode: elastase, cell-associated hemolysin, cell-free hemolysin, rhamnolipid, and alginate	[276]
	Inhibition of flagellin synthesis due to inhibition of *fliC* gene expression	[275]
	Inhibition of the production of acyl-hoserine lactone	[276]
